# An RNA thermometer dictates production of a secreted bacterial toxin

**DOI:** 10.1371/journal.ppat.1008184

**Published:** 2020-01-17

**Authors:** Christian Twittenhoff, Ann Kathrin Heroven, Sabrina Mühlen, Petra Dersch, Franz Narberhaus

**Affiliations:** 1 Microbial Biology, Ruhr University Bochum, Bochum, Germany; 2 Department of Molecular Infection Biology, Helmholtz Centre for Infection Research, Braunschweig, Germany; 3 Institute of Infectiology, Center for Molecular Biology of Inflammation, University of Münster, Münster, Germany; Collège de France, FRANCE

## Abstract

Frequent transitions of bacterial pathogens between their warm-blooded host and external reservoirs are accompanied by abrupt temperature shifts. A temperature of 37°C serves as reliable signal for ingestion by a mammalian host, which induces a major reprogramming of bacterial gene expression and metabolism. Enteric *Yersiniae* are Gram-negative pathogens accountable for self-limiting gastrointestinal infections. Among the temperature-regulated virulence genes of *Yersinia pseudotuberculosis* is *cnfY* coding for the cytotoxic necrotizing factor (CNF_Y_), a multifunctional secreted toxin that modulates the host’s innate immune system and contributes to the decision between acute infection and persistence. We report that the major determinant of temperature-regulated *cnfY* expression is a thermo-labile RNA structure in the 5’-untranslated region (5’-UTR). Various translational gene fusions demonstrated that this region faithfully regulates translation initiation regardless of the transcription start site, promoter or reporter strain. RNA structure probing revealed a labile stem-loop structure, in which the ribosome binding site is partially occluded at 25°C but liberated at 37°C. Consistent with translational control in bacteria, toeprinting (primer extension inhibition) experiments *in vitro* showed increased ribosome binding at elevated temperature. Point mutations locking the 5’-UTR in its 25°C structure impaired opening of the stem loop, ribosome access and translation initiation at 37°C. To assess the *in vivo* relevance of temperature control, we used a mouse infection model. *Y*. *pseudotuberculosis* strains carrying stabilized RNA thermometer variants upstream of *cnfY* were avirulent and attenuated in their ability to disseminate into mesenteric lymph nodes and spleen. We conclude with a model, in which the RNA thermometer acts as translational roadblock in a two-layered regulatory cascade that tightly controls provision of the CNF_Y_ toxin during acute infection. Similar RNA structures upstream of various *cnfY* homologs suggest that RNA thermosensors dictate the production of secreted toxins in a wide range of pathogens.

## Author summary

Bacterial pathogens closely survey the ambient conditions and induce virulence genes only at appropriate conditions. Upon host contact, many pathogens secrete toxins in order to subvert host defense systems. We find that such a secreted toxin in enteropathogenic *Yersinia pseudotuberculosis* is produced only at host body temperature. This regulation depends on a temperature-responsive RNA structure, an RNA thermometer, in the 5’-untranslated region of the toxin mRNA, which prevents translation at low temperatures when the bacterium is outside the host. Preventing melting of the RNA structure at 37°C by nucleotide substitutions that stabilize base pairing resulted in avirulent *Yersinia* strains unable to infect mice. Given that similar RNA thermometer-like structures exist upstream of related toxin genes in various bacterial pathogens, we propose that RNA thermometer-mediated toxin production is an evolutionary conserved mechanism. Interfering with opening of such regulatory structures might thus be a promising strategy targeting a broad spectrum of bacterial pathogens.

## Introduction

Throughout their infection cycle, bacterial pathogens encounter changing nutritional and physical conditions. To withstand these potentially harmful cues, pathogens constantly monitor multiple external and internal parameters. Temperature is among the most important signals upon entry into or exit from the host and induces profound changes in gene expression and metabolism [1]. Bacterial pathogens have evolved diverse signal transduction cascades that adjust gene expression as soon as they are confronted with elevated temperatures (37°C) in the warm-blooded host. Such a shift in temperature has an impact on almost any biomolecule. For example, it influences the activity or conformation of proteins and their interaction with membrane lipids. Moreover, it can affect the spatial arrangement of DNA and the structure of RNA [1–4]. The latter is particularly relevant in RNA thermometers (RNATs) that rearrange their structure in a temperature-dependent manner and thereby control translation efficiency. They are found in the 5’-untranslated region (5’-UTR) or intercistronic regions (ICRs) of many bacterial mRNAs [5,6]. At low temperatures, a secondary structure occludes the Shine-Dalgarno (SD) sequence and/or the translation initiation codon leading to inhibition of ribosome binding and, ultimately, inhibition of translation. An increase in ambient temperature provokes a gradual melting of the secondary structure, which liberates the ribosome-binding site (RBS) enabling translation initiation. Because of their instantaneous and reversible zipper-like control mechanism, RNATs are well-suited to adjust expression of virulence genes in bacterial pathogens during their infection cycle [7,8].

Besides *Yersinia pestis* and *Yersinia enterocolitica*, the Gram-negative γ-proteobacterium *Yersinia pseudotuberculosis* is one of the three pathogenic species from the genus *Yersinia*. While infection with *Y*. *pestis*, the causative agent of plague, leads to a grave and rapidly progressing febrile illness with severe mortality rates, the enteric foodborne pathogens *Y*. *pseudotuberculosis* and *Y*. *enterocolitica* cause self-limiting gastrointestinal diseases such as enteritis, diarrhea, and mesenteric lymphadenitis [9,10]. To infect target tissues and to survive in them, pathogenic *Yersinia* species possess an armory of virulence factors. Among these are the type three secretion system (T3SS), the *Yersinia* outer proteins (Yop effectors), and the adhesin YadA [11], whose genes are tightly controlled in response to extracellular calcium (Ca^2+^) concentration [12,13] and temperature [14]. Transcription of these virulence genes is activated by the low calcium response regulator (LcrF) which underlies an extensive thermoregulation cascade in *Y*. *pestis* and *Y*. *pseudotuberculosis* [15,16]. An RNAT in the *yscW-lcrF* intercistronic region plays a key role in this process as it mediates translational control of *lcrF* expression [15].

Previously, we experimentally mapped the structures of more than 1,750 *Y*. *pseudotuberculosis* RNAs by applying the parallel analysis of RNA structure (PARS) method [17] at three physiologically important temperatures. We were particularly interested in Shine-Dalgarno (SD) regions that unfold upon temperature elevation and identified at least 16 new RNATs controlling translation of genes from different functional categories [18]. A virulence-related thermometer candidate was found in the 5’-UTR of *cnfY* encoding the cytotoxic necrotizing factor (CNF_Y_). In contrast to many other cell-targeting *Yersinia* virulence factors, CNF_Y_ is not delivered via the T3SS but through extracellular membrane vesicles [19]. Once inside the host cell, this single-chain A-B toxin modulates the immune response by constitutively activating Rho GTPases and enhancing delivery of Yop effector proteins into target cells [20–22]. CNF_Y_ activity ultimately leads to the induction of acute inflammatory responses and to the formation of necrotic areas in infected tissues [20]. Related CNFs were identified in uropathogenic *Escherichia coli* (UPEC) strains (CNF1-3) as well as in *Y*. *pseudotuberculosis* strains YPIII and IP2666 [23,24].

In this study, we characterized the thermoregulatory features of the *cnfY* RNAT. We show that it forms a new type of structure, which is thermo-labile and allows increased ribosome binding at body temperature. Point mutations that strengthen the anti-SD/SD interaction prevent CNF_Y_ production at 37°C. *Y*. *pseudotuberculosis* strains carrying such stabilized RNAT variants were avirulent in a mouse model demonstrating the importance of precise temperature sensing for successful host-microbe interactions.

## Material and methods

### Ethics statement

Animal work was performed in strict accordance with the European Health Recommendations of the Federation of Laboratory Animal Science Associations (FELASA). The protocol was approved by the Niedersächsisches Landesamt für Verbraucherschutz und Lebensmittelsicherheit: animal licensing committee permission no. 33.19-42502-04-18/3034. Animals were treated with appropriate care and all efforts were made to minimize suffering.

### Bacterial strains and plasmids

Bacterial strains used in this study are listed in S1 Table. Cells were grown in LB medium or BHI medium (Sigma-Aldrich, St. Louis, USA) at indicated temperatures. If required, antibiotics were added to the media at following final concentrations: ampicillin, 100 μg/ml; gentamycin, 10 μg/ml; kanamycin, 50 μg/ml. For the induction of the P_*BAD*_ promoter the medium was supplemented with L-arabinose to a final concentration of 0.01% (w/v; *E*. *coli*) or 0.1% (w/v; *Y*. *pseudotuberculosis*). To generate YP216, the kanamycin resistance of strain YP147 was removed as described previously [25].

### Plasmid construction

All utilized oligonucleotides and plasmids are summarized in S2 or S3 Tables, respectively. Enzymes for cloning were obtained from Thermo Scientific (St. Leon-Rot, Germany). PCR, DNA manipulations, DNA restrictions, and transformations were performed according to standard protocols [26]. Point mutations were generated by site-directed mutagenesis according to the instruction manual of the QuikChange mutagenesis kit (Agilent Technologies, Santa Clara, USA). All constructed plasmids were confirmed by restriction analysis and automated sequencing (Eurofins Genomics, Ebersberg, Germany).

The *cnfY* RNAT:*gfp* fusion plasmid pBO4481 was constructed as follows: Since the *cnfY* 5’-UTR contains an internal *Eco*RI restriction site, a 128 bp fragment, including the *cnfY* 5’-UTR (mutation TT48,49AA, deleting the internal *Eco*RI site) and 30 bp of the *cnfY* coding region, was PCR-amplified using primer pair YPK_2615utr_fw/YPK_2615utr_rv and pBO3190 as template. The resulting DNA fragment was ligated into the *Eco*RI/*Nhe*I restriction sites of pBAD2-*gfp* obtaining pBO4478. Subsequently, the mutation (TT48,49AA) was restored by site-directed mutagenesis using primer pair YPK2615_QC2_fw/YPK2615_QC2_rv, resulting in plasmid pBO4481. Mutations R1 (AG32-33CT), R2 (A29Δ), and R1+2 (ATCAG29-33TCCT) were inserted into pBO4481 by site-directed mutagenesis using primer pairs cnfY_R1_fw/cnfY_R1_rv, cnfY_R2_fw/cnfY_R2_rv, and cnfY_R1+2_fw/cnfY_R1+2_rv generating plasmids pBO6505, pBO6506, and pBO6507, respectively. Plasmid pBAD2-*lcrF*-*gfp* (pBO4477) was constructed by integration of a 148 bp, PCR-amplified (primer pair lcrF_ICR_fw/lcrF_ICR_rv) DNA fragment into the *Eco*RI/*Nhe*I restriction sites of pBAD2-*gfp*. Plasmid pBO6528 was engineered as follows: First, an *Eco*RI restriction site was replaced by a *Sac*I site via site-directed mutagenesis (primer pair bgaB_Eco_Sac_fw/ bgaB_Eco_Sac_rv) resulting in pBO6524. Then, the YPK_2615 (*cnfY*) 5’-UTR (harbors two *Eco*RI restriction sites) was PCR-amplified (312 bp) with primer pair cnfY_TSSl_fw/cnfY_TSSl_rv and ligated into *Nhe*I/*Sac*I restriction sites of pBO6524 obtaining pBO6527. The *Sac*I restriction was exchanged by a *Eco*RI site using site-directed mutagenesis (primer pair cnfY_Sac_Eco_fw/cnfY_Sac_Eco_rv) resulting in plasmid pBO6528. Plasmid pBO6523 was constructed applying site-directed mutagenesis (T26C) with primer pair cnfY_Ype_fw/cnfY_Ype_rv. Mutation R1+2 (ATCAG29-33TCCT) was inserted into pBAD2-*cnfY*-*bgaB*-His (pBO3192) via site-directed mutagenesis (primer pair cnfY_R1+2_fw/cnfY_R1+2_rv) obtaining pBO4449.

The run-off plasmid for *in vitro* transcription the *cnfY* RNAT (pBO4465) was constructed by blunt-end ligation of a PCR-amplified DNA fragment (primer pair cnfY_RO_fw/cnfY_RO_rv), comprising the T7 RNA polymerase promoter, and the *cnfY* RNAT including 80 bp of the *cnfY* coding region, into the *Sma*I restriction site of pUC18. Insertion of the repressive R1+2 mutation (ATCAG29-33TCCT) into pBO4465 was achieved by site-directed mutagenesis (primer pair cnfY_R1+2_fw/cnfY_R1+2_rv), resulting in pBO4466. Plasmids pBO4493 and pBO4494 were created by Eurofins Genomics (Ebersberg, Germany). Briefly, two DNA fragments, comprising the promoter region, the 5’-UTR as well as 123 bp of the coding region of *cnfY*, were synthesized. These fragments were ligated into pEX-K168 (Eurofins Genomics, Ebersberg, Germany) and include the repressive mutations R1 (AG32-33CT) or R1+2 (ATCAG29-33TCCT), respectively. For construction of the translational gene fusions to *luxCDABE*, the promoter region, the 5’-UTR (wild type, R1, and R1+2 variant of the *cnfY* RNAT) as well as 30 bp of the coding region of *cnfY* were PCR-amplified (GmR_NcoI_fw/GmR_BamHI_rv)), using genomic DNA, pBO4493, and pBO4494, as template. Next, the resulting DNA fragments were ligated via *Bam*HI/*Sal*I restriction sites into pFU53, obtaining plasmids pBO6500, pBO6501, and pBO6502, respectively. The construction of the *cnfY* complementation plasmids, harboring the *cnfY* promoter region and the repressive *cnfY* RNAT variants R1 (AG32-33CT; pBO6503) or R1+2 (ATCAG29-33TCCT; pBO6504) was carried out as follows. First, a gentamycin resistance cassette was PCR-amplified, using pBAD2-*gfp* as template, and ligated into pJNS10 via *Bam*HI/*Nco*I restriction sites, obtaining pJNS10 ΔP_*cnfY*_::Gen^R^ (pBO4499). Next, two fragments comprising the promoter region, the 5’-UTR as well as 123 bp of the coding region of *cnfY* were PCR-amplified (primer pair PcnfY_BamHI_fw/cnfY_NcoI_rv) from pBO4493 and pBO4494, respectively. Finally, these fragments were integrated into the *Bam*HI/*Nco*I restriction sites of pBO4499 obtaining plasmids pBO6503 and pBO6504, respectively.

### Reporter gene activity assays

For β-galactosidase activity assays, *E*. *coli* DH5α or *Y*. *pseudotuberculosis* YPIII cells carrying the *cnfY* 5’-UTR:*bgaB* fusion plasmids were grown overnight in LB with ampicillin at 25°C. Before being inoculated with an overnight culture (OD_600_ = 0.2), LB media supplemented with ampicillin was pre-warmed to 25°C. After growth to an OD_600_ = 0.5, transcription was induced with 0.01% w/v (*E*. *coli*) or 0.1% w/v (*Y*. *pseudotuberculosis*) L-arabinose. The culture was split up and shifted to pre warmed 100 ml flasks (temperatures indicated in the respective figure). The cultures were incubated for 30 min and 400 μl samples were subsequently taken for β-galactosidase assay. The β-galactosidase assay was carried out as described previously [18,27]. Standard deviations were calculated from three technical replicates.

The *gfp* reporter gene assay was performed as follows: *E*. *coli* DH5α or *Y*. *pseudotuberculosis* YPIII cultures carrying the wild type of the mutated *cnfY* 5’-UTR:*gfp* fusion plasmids were grown and treated as described above. Afterwards, 2 ml samples were taken for Western blot analysis and 4 ml samples were taken for total RNA isolation and subsequent Northern blot analysis.

### Western blot analysis

Cell pellets of *E*. *coli* DH5α, *Y*. *pseudotuberculosis* YPIII, *Y*. *pseudotuberculosis* YP216 were resuspended in 1 x SDS sample buffer (2% (w/v) SDS, 0.1% (w/v) bromophenol blue, 10% glycerol, 50 mM Tris/HCl, pH 6.8) according to their optical density (CNF_Y_ synthesis studies: 100 μl for OD_600_ = 1; *gfp* reporter gene assay: 100 μl for OD_600_ = 0.5). After boiling for 10 min at 95°C, samples were centrifugated (10 min, 13000 rpm) and the protein extracts were subjected to SDS gel electrophoresis (10–12.5% SDS PAA gels). Subsequent Western transfer was performed by tank blotting onto a nitrocellulose membrane (Hybond-C Extra, GE Healthcare, Munich, Germany). An anti-CNF_Y_ antibody was used in a 1:1000 dilution, anti-GFP antibody (ABIN129570, antibodies-online GmbH, Aachen) was used in a 1:10000 dilution and secondary antibody goat anti-rabbit-HRP conjugate (Bio-Rad, Munich, Germany) in a 1:3000 dilution. Luminescence signals were detected by incubating membranes with Luminata Forte Western HRP (Merck, Darmstadt, Germany) substrate and the ChemiImager Ready (Alpha Innotec, San Leandro, USA).

### RNA isolation

Pellets of *E*. *coli* DH5α or *Y*. *pseudotuberculosis* YPIII cells were washed with 1 ml cold resuspension buffer (10 mM TRIS/HCl pH 8.0, 100 mM NaCl, 1 mM EDTA). After resuspension in 250 μl cold resuspension buffer and addition of 250 μl lysis buffer (50 mM TRIS/HCl pH 8.0, 8% (w/v) sucrose, 1% (v/v) Triton X-100, 0.4% (w/v) lysozyme, 10 mM EDTA), cells were heated to 65°C for 30 s. RNA was obtained from samples by four times extraction with acid phenol followed by three times extraction with chloroform/isoamylalcohol (24:1). After ethanol precipitation, RNA pellets were resuspended in 50 μl ddH_2_O. RNA concentration was measured with a NanoDrop spectrophotometer ND-1000 (PEQLAB Biotechnologie GmbH, Erlangen, Germany).

### Northern blot analysis

Northern blot analysis was performed as described previously [28]. For detection of *gfp* transcript a 286 bp fragment was PCR-amplified from pBAD2-*gfp*. RNA probes were derived from *in vitro* transcription with digoxigenin (DIG) labeled nucleotides (Roche, Mannheim, Germany).

### Quantitative real-time PCR (qRT-PCR)

Samples for comparative qRT-PCR and Western blot analysis of *cnfY* expression were taken from the same cultures. Transcript abundance was determined from three independent cultures measured in duplicate. The non-thermoregulated reference genes *gyrB* and *nuoB* were used for normalization [29]. After isolation, 10 μg total RNA were treated with DNase (DNA-free Kit, Invitrogen, Carlsbad, United States) followed by cDNA synthesis (iScript cDNA synthesis Kit, Bio-Rad, Munich, Germany) according to the manufactures protocols (1 μg RNA per reaction). Two μl of diluted cDNA (1:10) were mixed with 0.25 μl of each primer (10 ρM), 5 μl of iTaq Universal SYBR Green Supermix, and 2.5 μl sterile water (Carl Roth GmbH, Karlsruhe, Germany). Amplification and detection were performed in a CFX Connect Real-Time System (Bio-Rad, Hercules, United States) under conditions optimized for each gene target. For analysis of each gene target, a six-point standard curve was included to ensure satisfactory amplification efficiency and ascertain that all samples fall within the linear range of the standard curve. Relative *cnfY* transcript amounts were calculated using the ΔΔCt method [30] with experimental Ct values normalized to that of *nuoB* and *gyrB*. Primer efficiencies calculated by the CFX Maestro software were as follows: *gyrB*: 102.9%, *nuoB*: 102.7%, and *cnfY*: 98.4%.

### *In vitro* transcription

RNAs for structure probing and primer extension inhibition experiments were synthesized *in vitro* by run-off transcription with T7 RNA polymerase (Thermo Scientific, St. Leon-Rot, Germany) from *Eco*RV-linearized plasmids (listed in S3 Table) as previously described [18].

### Enzymatic structure probing

Structure probing of the 5’-UTR and 80 nt of *cnfY* was performed with *in vitro* transcribed RNA using pBO4465 or pBO4466 as template. The *in vitro* transcribed RNA was purified and dephosphorylated using CIP enzyme (Calf intestinal phosphatase, Thermo Scientific, Waltham, USA). The RNA was labeled with [^32^P] at the 5′ end as described elsewhere [31]. Partial digestions of radiolabeled RNA with ribonuclease T1 (0.0025 U) (Thermo Scientific, Waltham, USA) and T2 (0.056 U) (MoBiTec, Göttingen, Germany) were performed according to [32] at 25, 37, and 42°C. For digestion with RNase T1 and RNase T2 a 5 x TN buffer (100 mM Tris acetate, pH 7, 500 mM NaCl) was used. An alkaline hydrolysis ladder was prepared as described before [31]. The T1-ladder was generated by using 30000 cpm labeled RNA. The RNA was heated with 1 μl sequencing buffer (provided with RNase T1) at 90°C. Afterwards, the RNA was incubated with the enzyme at 37°C for 5 min.

### Primer extension and Primer extension inhibition analysis (toeprinting)

The *cnfY* transcriptional start site was mapped by primer extension as described previously [33]. Primer cnfY_RO_rv was used for reverse transcription of RNA isolated from bacteria that were grown at 37°C. The DNA sequence reaction was performed with the same primer using the Thermo Sequenase cycle sequencing kit (USB, now Affymetrix) and plasmid pBO4465 as template.

Toe printing analysis was performed with 30S ribosomal subunit, *in vitro* transcribed RNA and tRNA^fMet^ (Sigma-Aldrich, St. Louis, MO, United States) according to a protocol described before [34]. A 5’-[^32^P]-labeled *cnfY*-specific oligonucleotide cnfY_RO_rv, complementary to nucleotides +61 to +80 (from ATG) of the *cnfY* mRNA, was used as a primer for cDNA synthesis. The radiolabeled primer (0.16 ρmol) was annealed to the *cnfY* mRNA (0.08 ρmol) and incubated with 30S ribosomal subunit (24 ρmol) or Watanabe buffer (60 mM HEPES/KOH; 10.5 mM Mg(COO)_2_; 690 mM NH_4_COO; 12 mM β-mercaptoethanol; 10 mM spermidine; 0.25 mM spermine) in presence of tRNA^fMet^ (8 ρmol) at 25, 37 or 42°C for 10 min. After addition of 2 μl MMLV-Mix (VD+Mg^2+^ buffer, BSA, dNTPs and 800 U MMLV reverse transcriptase (Affymetrix, Santa Clara, CA, US), cDNA synthesis was performed for 10 min at 37°C. Reaction was stopped by addition of formamide loading dye and samples were separated on a 8% denaturing polyacrylamide gel. The Thermo Sequenase cycle sequencing Kit (USB, now Affymetrix) was used for sequencing reactions with plasmid pBO4465 as template and radiolabeled primer cnfY_RO_rv.

### Measurement of *cnfY* expression by bioluminescence

*Y*. *pseudotuberculosis* YPIII harboring the fusion plasmids pBO6500, pBO6501, or pBO6502 were diluted 1:50 in fresh LB from overnight cultures and grown to late exponential phase (4 h) at 25°C or 37°C. Reporter fusions (transcriptional and translational fusions to *luxCDABE*) emitting bioluminescence were measured in non-permeabilized cells with a Varioskan Flash (Thermo Scientific, St. Leon-Rot, Germany) using the SkanIt software (Thermo Scientific, St. Leon-Rot, Germany) or with a CLARIOstar plus reader (BMG Labtech, Ortenberg, Germany). The data are given as relative light units (RLU/OD_600_) from three independent cultures measured in triplicate.

### Mouse infection studies

Female BALB/c mice (6- and 8-week-old) were acquired from Janvier (Saint Berthevin Cedex, France) and housed under specific pathogen-free conditions in accordance with FELASA recommendations in the animal facility of the Helmholtz Centre for Infection Research, Braunschweig.

For survival assays, mice were infected orally via food pellets inoculated with approximately 2x10^9^ bacteria of *Y*. *pseudotuberculosis* strains YPIII with the empty vector pJNS11 and YP147 (*cnfY*::*KanR*), harboring either the empty vector pJNS11 or the *cnfY* complementation vector pJNS10 (pP_*cnfY*_:*cnfY*^+^) with either the wild type, the R1, or the R1+2 variant of the *cnfY* RNAT. Prior to infection, bacterial cells were washed three times and resuspended in sterile PBS. The infected mice were monitored daily for 14 days to determine the survival rate, the body weight and health status. Bacteria used for organ burden experiments were grown over night in LB medium at 25°C, washed and resuspended in sterile PBS. Groups of 5 animals were infected orally (via inoculated food pellets) with approximately 2x10^8^ bacteria of *Y*. *pseudotuberculosis* strains YPIII with the empty vector pJNS11 and YP147 (*cnfY*::Kan^R^), harboring either the empty vector pJNS11 or the *cnfY* complementation vector pJNS10 (pP_*cnfY*_:*cnfY*^+^) including the wild type, the R1, or the R1+2 variant of the *cnfY* RNAT. Five days after infection, mice were euthanized by CO_2_ asphyxiation. The small intestine, the mesenteric lymph nodes, the liver, and the spleen were isolated and the Peyer’s patches were removed from the small intestine. Subsequently, whole organs were weighed and homogenized in PBS at 30000 rpm for 20 sec using a Polytron PT 2100 homogenizer (Kinematica, Switzerland). To determine the bacterial load of the organs, serial dilutions of the homogenates were plated on LB plates with 0.5 μg/ml triclosan. The colony forming units (cfu) were counted and are given as cfu per g organ/tissue.

## Results

### The *cnfY* transcript harbors a temperature-sensitive RNA element

**Fig 1:**
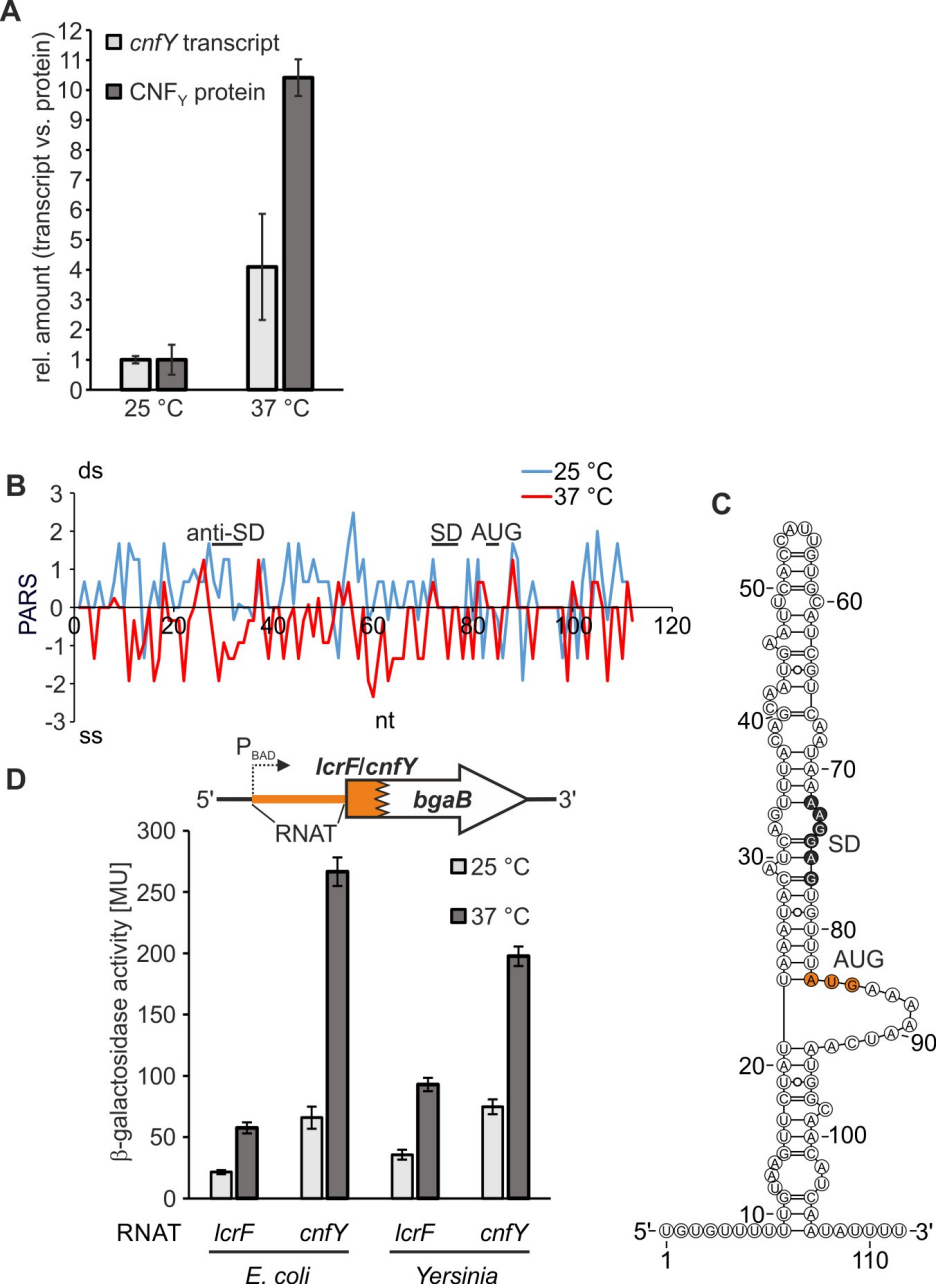
The *cnfY* 5’-UTR comprises a temperature-sensitive regulatory RNA element. (A) Comparison of *cnfY* transcript and CNF_Y_ protein levels at 25°C and 37°C. *Y*. *pseudotuberculosis* YPIII was grown to exponential growth phase (OD_600_ = 0.5) at 25°C or 37°C and samples were harvested for subsequent qRT-PCR and Western blot analysis. Temperature-dependent transcription was measured by qRT-PCR. Data were analyzed by the ΔΔCt method [30] and *cnfY* transcription was normalized to that of reference genes *nuoB* and *gyrB*. Amounts of *cnfY* transcript relative to levels detected at 25°C are shown. CNF_Y_ signals from Western blot analysis were quantified by integrated density quantification using AlphaEaseFC software. The mean density values and standard deviations from three independent experiments were calculated and normalized by the respective mean density value measured at 25°C. A representative Western blot is displayed in S1A Fig. (B) PARS profiles of the *cnfY* RNAT (-82 nt to +30 nt from AUG) at 25°C and 37°C. The potential SD sequence, its pairing sequence (anti-SD), and the AUG are marked. (C) PARS-derived secondary structure of *cnfY* (-82 nt to +30 nt from AUG) at 25°C [18]. The potential SD region and the translation start codon (AUG) are highlighted in black and orange, respectively. (D) The RNA control element in the *cnfY* 5’-UTR confers temperature-dependent reporter gene expression. To test for temperature-dependent translational control, the *cnfY* RNAT was translationally fused to *bgaB* under control of the P_*BAD*_ promoter (pBO3192). The *yscW*-*lcrF* intercistronic region (*lcrF* RNAT) served as positive control (pBO3146). A schematic representation of the reporter gene fusions is displayed. *E*. *coli* DH5α and *Y*. *pseudotuberculosis* YPIII cells harboring the corresponding plasmid (technical triplicates per each construct) were grown to an OD_600_ = 0.5 at 25°C. Afterwards, transcription was induced with 0.01% (w/v) (*E*. *coli*) or 0.1% (w/v) (*Y*. *pseudotuberculosis*) L-arabinose, respectively. The cultures were split immediately: One half remained at 25°C while the other was transferred into pre-warmed flasks at 37°C. After 30 min incubation, samples were taken for subsequent β-galactosidase assay. The displayed results represent the mean activities from three independent experiments (biological replicates). Mean standard deviations are indicated as error bars.

Comparative RNAseq-based transcriptome profiling of *Y*. *pseudotuberculosis* grown at 25°C and 37°C, representing environmental and host body temperature, respectively, revealed an about 7-fold induction of *cnfY* transcripts at elevated temperature indicating transcriptional thermoregulation of *cnfY* expression [29]. To determine the corresponding protein levels of this toxin, we performed Western blot analysis using an antibody against CNF_Y_ after growing *Y*. *pseudotuberculosis* YPIII at 25°C or 37°C (S1A Fig). Substantially elevated levels of CNF_Y_ were detected at 37°C compared to 25°C regardless of whether cells were grown to exponential or early stationary growth phase. To distinguish between transcriptional and translational control, we performed qRT-PCR and Western blot analyses using simultaneously collected samples from exponentially grown cells. We found a four-fold increase of *cnfY* transcript and 10-fold increase of CNF_Y_ protein (Fig 1A; representative Western blot in S1B Fig). This finding strongly suggests an additional layer of thermoregulation at post-transcriptional level.

Our previous global analysis of the *Y*. *pseudotuberculosis* RNA structurome [18] had provided evidence for translational temperature regulation through unfolding of the *cnfY* 5’-UTR from 25°C to 37°C (drop in PARS values in particular around the SD and anti-SD regions; Fig 1B). The PARS-derived secondary structure of the *cnfY* 5’-UTR constitutes a stem-loop structure including the SD sequence (5’-AAGGAG-3’) that partially pairs with a 5’-CAUCAGU-3’ region (Fig 1C). The intramolecular interaction region contains a bulged nucleotide (A29) and an internal loop (AG32-33, AG73-74) in the anti-SD/SD region, which are typical characteristics of temperature-sensitive RNA structures able to melt at elevated temperatures. The AUG translation start codon is part of a 9-nt loop, which separates the stem-loop in a short basal and a long terminal part. The overall sequence and structure is unrelated to the fourU-type RNAT upstream of *Yersinia lcrF* and does not resemble any of the known RNAT families.

Preliminary experiments in *E*. *coli* suggested that the *cnfY* 5’-UTR is a translational control element [18]. To characterize the regulatory features further, we established a new protocol for measuring *bgaB*-derived β-galactosidase activity in *Y*. *pseudotuberculosis* at 25°C and 37°C. The well-studied *lcrF* RNAT served as positive control [15]. The *cnfY* RNAT showed a clear induction of *bgaB* activity from 25 to 37°C comparable to the positive control and regardless of whether being tested in *E*. *coli* or *Y*. *pseudotuberculosis* (Fig 1D). Overall, these results show that *cnfY* expression is under combined transcriptional [29] and translational control.

### Thermosensing by the *cnfY* RNAT is independent of the transcription start site

**Fig 2:**
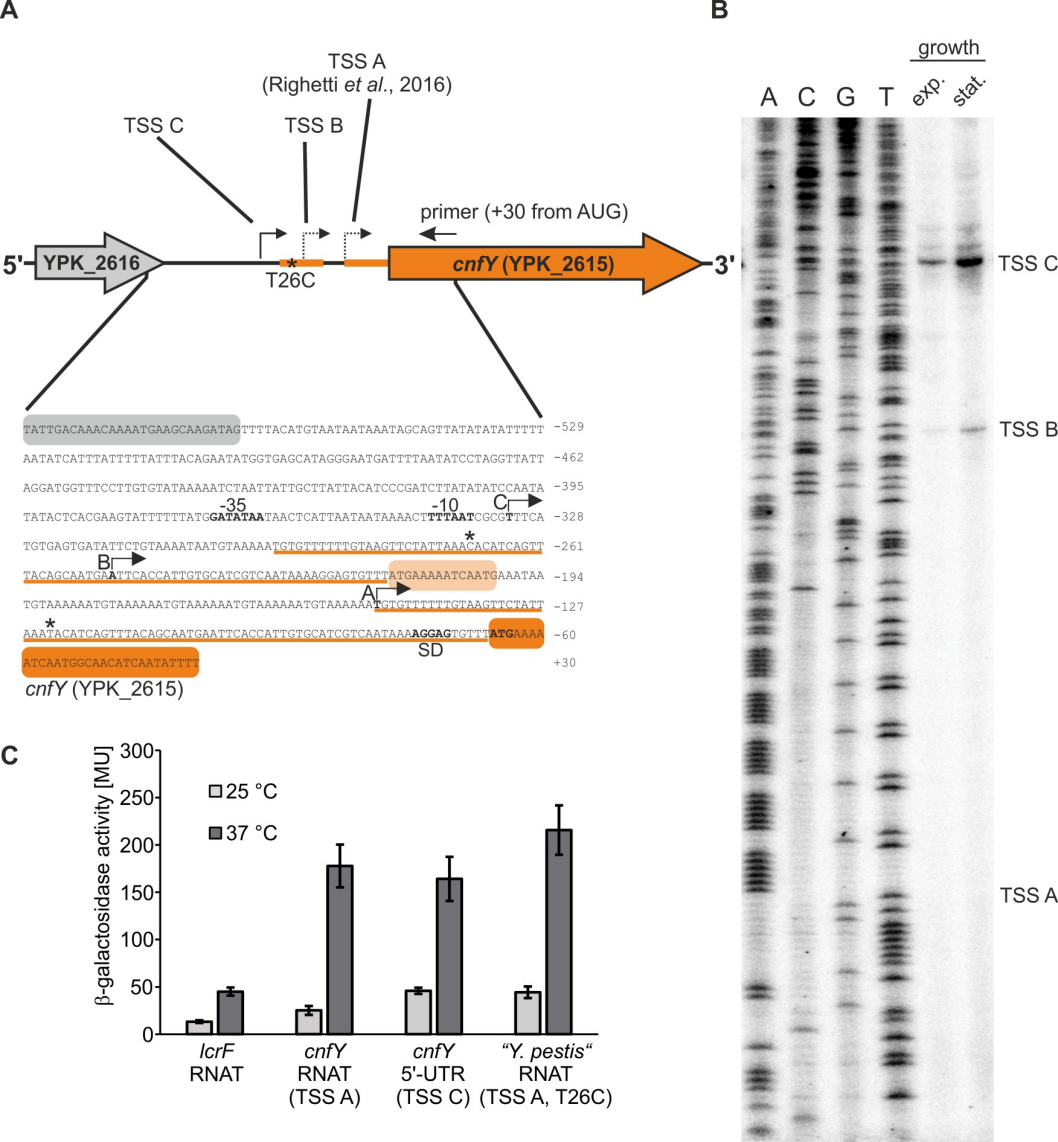
The *cnfY* RNAT functions regardless of the transcriptional site. (A) Schematic representation of the *cnfY* (YPK_2615) gene locus. Transcriptional start sites (TSS) identified in a previous study [18] and in this study are marked by arrows. Additionally, the sequence of the intergenic region between YPK_2616 (coding for a putative transposase) and *cnfY* is displayed. The underlined sequences (orange line) represent the duplicated RNAT sequences. The sequence variation T26C observed in the duplicated sequences is marked by asterisks. (B) Identification of *cnfY* TSSs by primer extension. Total RNA was isolated from a *Y*. *pseudotuberculosis* YPIII culture grown at 37°C to exponential (OD_600_ = 0.5) and stationary growth phase (OD_600_ = 1.5). Primer extension was performed using a radiolabeled primer (binding is shown in A). The cDNA products were separated via polyacrylamide gel electrophoresis. The experiment was conducted in three biological replicates. (C) *Y*. *pseudotuberculosis cnfY* 5’-UTRs (+30 nt of the coding region; pBO3192, pBO6527) and the *Y*. *pestis cnfY* RNAT (-82 nt to +30 nt from AUG; pBO6523) were translationally fused to *bgaB* and tested for β-galactosidase activity. The *lcrF* RNAT was used as positive control (pBO3146). *E*. *coli* DH5α cells harboring the corresponding plasmid (technical triplicate per each construct) were grown to an OD_600_ = 0.5 at 25°C. Afterwards, transcription was induced with 0.01% (w/v) L-arabinose and the culture was split. One half remained at 25°C while the other was transferred into pre-warmed flasks (37°C). After 30 min of incubation, samples were taken for subsequent β-galactosidase assay. The displayed results represent the mean activities from three independent experiments (biological replicates). Mean standard deviations are indicated as error bars.

Based on transcriptional profiling data, we previously assumed a transcriptional start site (TSS) of *cnfY*, which is located 82 nt upstream of the AUG start codon (TSS A; Figs S2A and 2A). The corresponding 5’-UTR is able to confer thermal control in a reporter gene assay ([18] and Fig 1D). Two observations made us reassess the TSS. First, two additional peaks in an earlier transcriptome profile by Nuss et al. ([29] and S2A Fig) and secondly, a striking almost identical duplication further upstream of the RNAT in the *Y*. *pseudotuberculosis* YPIII genome (S2A Fig and Fig 2A). A transcript starting from TSS A covers a full-length copy of the RNAT. The duplicated RNAT sequence further upstream of TSS A deviates in one nucleotide (T26C).

To map the 5’-end(s) of the *cnfY* mRNA, we performed primer extension experiments on total RNA isolated from *Y*. *pseudotuberculosis* YPIII grown at 37°C. No signal was detected around position -82 relative to the AUG codon (TSS A, Fig 2A and 2B). Instead, we found a weak signal 183 nt (TSS B) and a strong signal 266 nt (TSS C) upstream of the start codon, respectively. The TSS C signal corresponds to the first of three signals visible in transcriptome profiling data of *Y*. *pseudotuberculosis* YPIII grown to exponential phase at 37°C [29] (S2A Fig). To test whether the long 5’-UTR is capable of regulating reporter gene expression in response to temperature, we compared the activities of P_*BAD*_ promoter-controlled translational *bgaB* fusions comprising the long TSS C or the short TSS A-derived 5’-UTRs followed by 30 nt of the coding region of *cnfY* fused to *bgaB* [18]. Both the long and short 5’-UTR conferred similar temperature-regulated expression (Fig 2C) suggesting that the TSS A-derived short 5’-UTR of *cnfY* with a single RNAT copy is sufficient for temperature regulation. From here on we will refer to this 5’-UTR (-82 nt from AUG) as *cnfY* RNAT.

Sequence comparison of the *cnfY* locus revealed duplicated thermometer sequences in other *Y*. *pseudotuberculosis* strains and in *Y*. *pestis* (S2B Fig). Interestingly, the nucleotide substitution (T26C) present in the distal copy of the thermometer in *Y*. *pseudotuberculosis* YPIII was also found in the downstream copy in several *Y*. *pseudotuberculosis* strains and in *Y*. *pestis* (S2B Fig). In the secondary RNA structure, nucleotide 26 pairs with G79 (Fig 1C) and the U to C exchange should strengthen this interaction. To analyze the impact of this single-nucleotide polymorphism (SNP) on translational control, a translational *bgaB* fusion of the U26C variant was constructed (“*Y*. *pestis*” RNAT in Fig 2C) and found to respond to increasing temperature like the *Y*. *pseudotuberculosis* YPIII thermometer demonstrating that the SNP-containing natural variants retained their regulatory potential.

### Point mutations impair thermometer functionality

**Fig 3:**
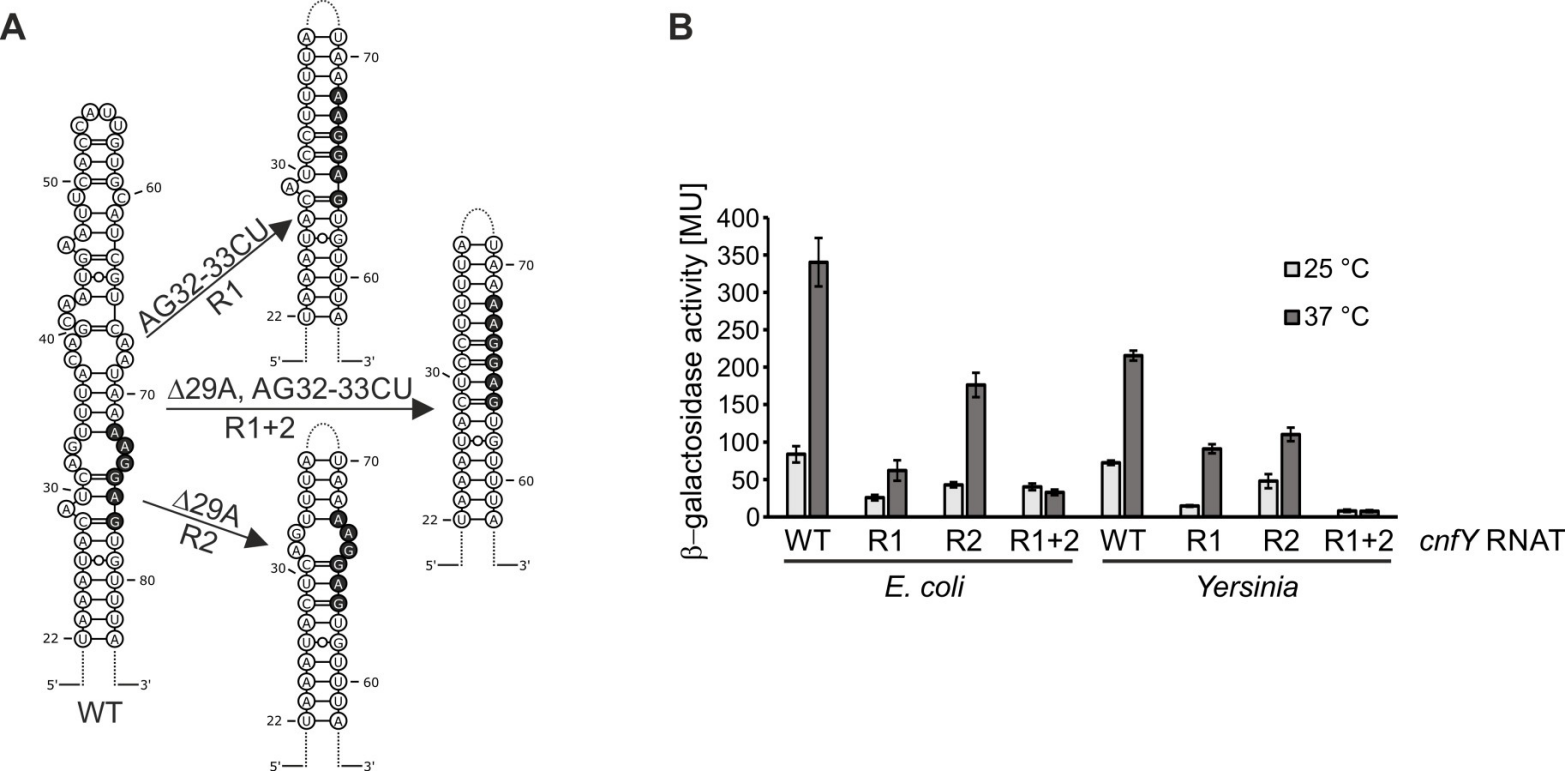
Stabilizing point mutations within the *cnfY* RNAT impair thermometer functionality. (A) Schematic representation of the PARS-derived secondary structure of the *cnfY* RNAT stem (nucleotides 22 to 83) including the SD region (black nucleotides). The predicted stabilized structures resulting from nucleotide exchanges are depicted to the right. (B) The wild type (WT: pBO4481) and the mutated *cnfY* RNATs (R1: pBO4610; R2: pBO4611; R1+2: pBO4449) were translationally fused to *bgaB* and tested for β-galactosidase activity as described in the legend to Fig 1D. The results represent the mean activities from three independent experiments (biological replicates) each done in triplicate. Mean standard deviations are indicated as error bars.

The naturally occurring SNPs in the *cnfY* 5’-UTR motivated us to construct RNAT variants with increased stability. The bulged A29 residue and the internal loop (AG32-33, AG73-74) in the antiSD/SD region are destabilizing elements (Fig 3A) and most likely critical for temperature-dependent regulation of *cnfY*. Using site-directed mutagenesis, we previously constructed the repressive (stabilizing) mutations R1 (AG32-33CU) and R2 (ΔA29) into the *cnfY* RNAT, which partially inhibited expression in the β-galactosidase assay in *E*. *coli* [18]. To achieve full repression, we combined both mutations to obtain the R1+2 (ΔA29, AG32-33CU) variant of the *cnfY* RNAT with a perfectly paired SD region (Fig 3A). The R1, R2, and R1+2 variants fused to *bgaB* were analyzed at 25°C and 37°C in *E*. *coli* and *Y*. *pseudotuberculosis* (Fig 3B). RNAT variants with mutations R1 (AG32-33CU) and R2 (ΔA29) exhibited reduced levels of reporter gene activity compared to the wild-type thermometer. The combined mutation R1+2 (ΔA29, AG32-33CU) impaired temperature sensing and resulted in an almost complete loss of β-galactosidase activity even at 37°C. Due to the similar responses in the heterologous *E*. *coli* host and in *Y*. *pseudotuberculosis*, we conducted all subsequent experiments in *Yersinia* only.

### The *cnfY* thermosensor controls translation in a temperature-dependent manner

**Fig 4:**
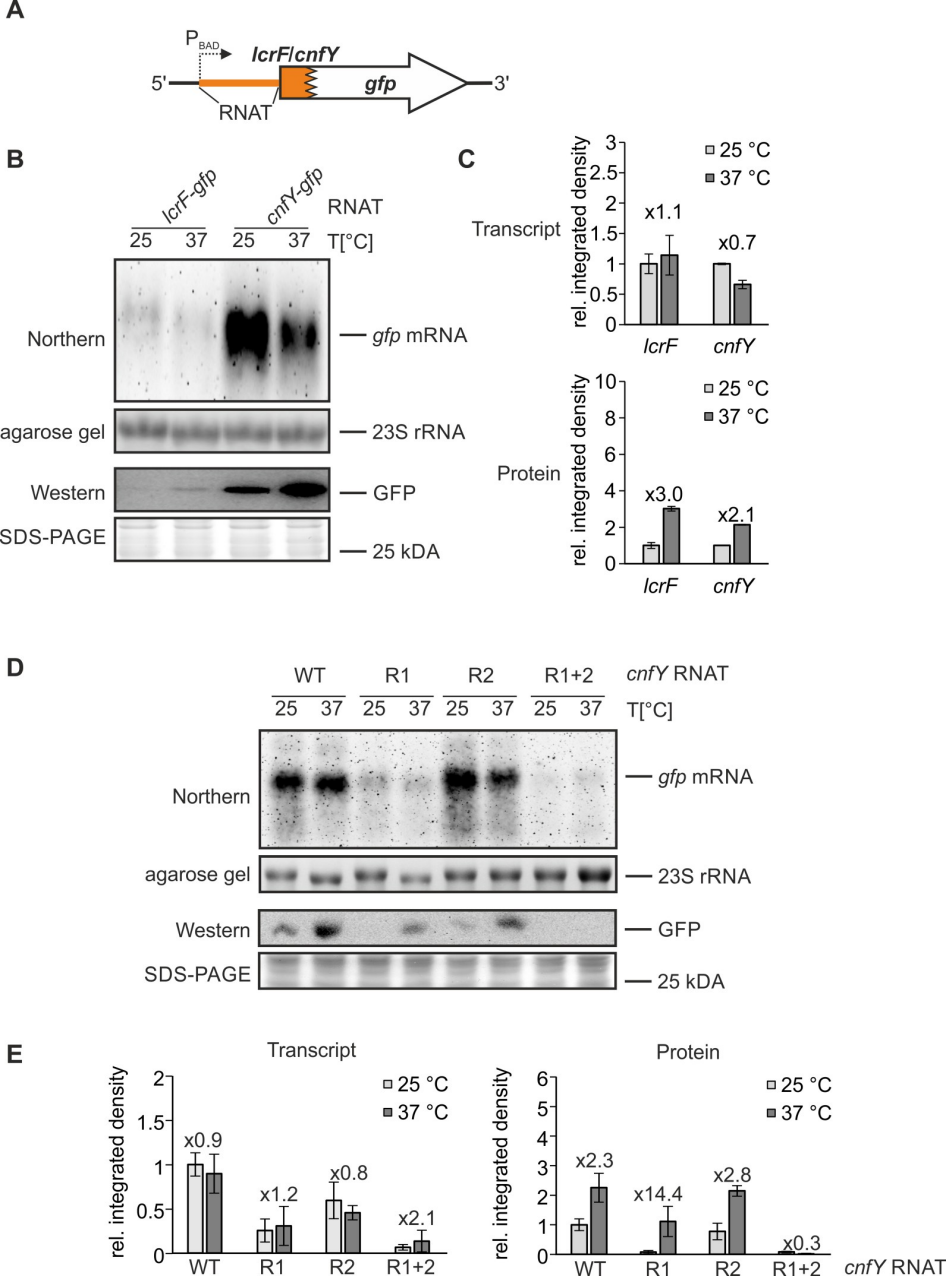
The *cnfY* RNAT facilitates translational thermoregulation of *gfp* expression. (A) Schematic representation of the translation *cnfY*:*gfp* reporter gene fusion on plasmid pBAD2-*cnfY*-*gfp* (pBO4481). (B) Cellular *gfp* transcript and GFP protein levels were measured by Northern and Western blot analyses, respectively. The *lcrF* RNAT translationally fused to *gfp* served as positive control (pBO4477). *Y*. *pseudotuberculosis* YPIII cells harboring the corresponding plasmids (technical triplicate per each construct) were grown to an OD_600_ = 0.5 at 25°C before transcription was induced with 0.1% (w/v) L-arabinose and the cultures were split. One half remained at 25°C while the other was transferred into pre-warmed flasks (37°C). After 30 min of incubation, samples were taken for Western and Northern blot analysis. (C) Densitometric quantification of signals from the Northern and Western blot experiments by the AlphaEaseFC software. The given values represent mean band intensities (37°C/25°C) and the corresponding standard deviations (three independent experiments). (D) The wild type (pBO4481) and the mutated *cnfY* RNATs (R1: pBO6505, R2: pBO6506, R1+2: pBO6507) were translationally fused to *gfp* and tested for differential GFP synthesis and *gfp* transcription in *Y*. *pseudotuberculosis* YPIII as described in Fig 3. Each experiment was performed at least in triplicate. (E) Comparison of transcriptional and translational control by the *cnfY* RNAT variants measured by densitometrical analysis. Signals from Northern and Western blot analysis were quantified by integrated density quantification using AlphaEaseFC software. The mean density values and its standard deviations (three independent experiments) were calculated and normalized by the respective mean density value measured for the wild type thermometer at 25°C.

To determine whether the *cnfY* RNAT mediates thermoregulation at translational rather than at transcriptional level of gene expression, we used a previously established *gfp*-based reporter gene system [35], in which the *cnfY* RNAT downstream of the P_*BAD*_ promotor is translationally fused to *gfp* (Fig 4A). We measured *gfp* transcript levels by Northern blots with an anti-*gfp* RNA probe and GFP protein by Western blots with a GFP-specific antibody after growing *Y*. *pseudotuberculosis* YPIII cultures at 25°C or after a shift to 37°C. A translational *lcrF* RNAT:*gfp* fusion served as positive control (Fig 4B). While reporter gene transcript levels remained nearly unaffected by temperature (Fig 4C), protein levels increased substantially after a shift to 37°C (Fig 4C). In accordance with the results of the β-galactosidase activity assay (Fig 1D), these results show that the *cnfY* RNAT confers temperature-dependent translational control to its downstream gene.

We also verified that the repressive mutations affected translation rather than transcription and determined RNA and protein levels by the pBAD2-*gfp* reporter system [35] as described above. In all cases, the transcript levels were similar at 25 and 37°C whereas the protein amounts of the WT, R1 and R2 fusions increased at 37°C (Fig 4D and 4E). Consistent with previous reports, the transcript levels of poorly translated repressive RNAT variants was low presumably because they are “naked” and not protected by translating ribosomes [28,36]. As observed with the *bgaB* fusions (Fig 3B), the R1 and R2 constructs retained some temperature responsiveness (Fig 4D and 4E). More specifically, stabilization of the SD region in R1 (Fig 3A) resulted in stronger translational repression compared to deletion of the bulged A in the anti-SD region in R2. The R1+R2 fusion combining both mutations was fully repressed.

### Thermosensing by the *cnfY* RNAT is independent of the promoter

**Fig 5:**
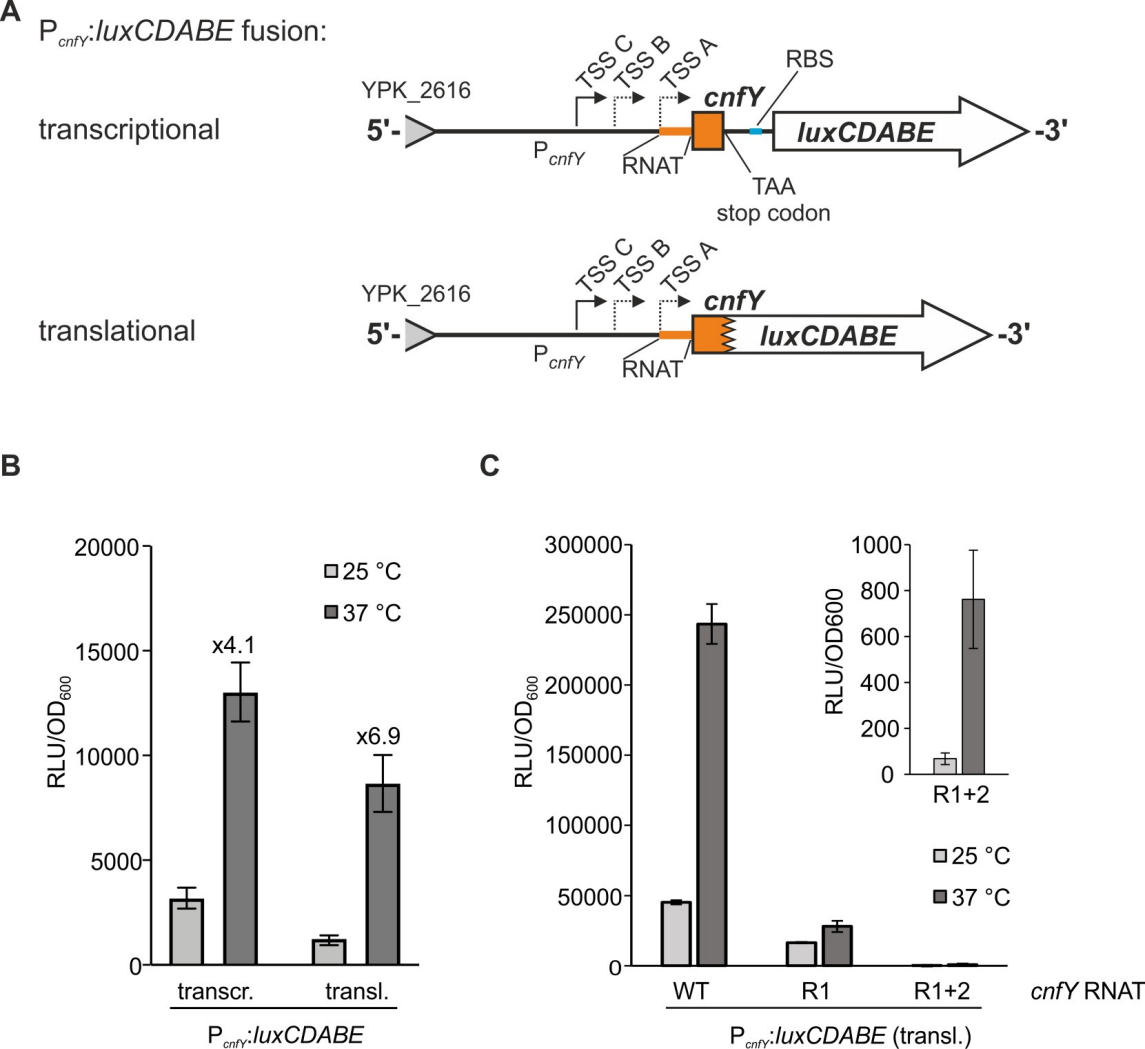
*In vivo* expression of P_*cnfY*_:*luxCDABE* fusions. (A) Schematic representation of a transcriptional (PJNS02, [20]) and a translational fusion (pBO6500) of the *cnfY* promoter region (P_*cnfY*_) to the *luxCDABE* reporter gene operon. (B) *Y*. *pseudotuberculosis* YPIII cultures harboring these plasmids were grown at 25°C and 37°C. Samples for bioluminescence measurement (described in Material and Methods) were taken after 4 hours. The data are depicted as relative light units (RLU/OD_600_) from three independent cultures measured on a CLARIOstar plus reader (BMG Labtech, Ortenberg, Germany). (C) *Y*. *pseudotuberculosis* YPIII cultures harboring the translational fusion including the wildtype (pBO65000), the R1 (pBO6501), or the R1+2 (pBO6502) RNAT variant were grown at 25°C and 37°C. Samples for bioluminescence measurement (described in Material and Methods) were taken after 4 hours. The data are depicted as relative light units (RLU/OD_600_) from three independent cultures measured on a Varioskan Flash (Thermo Scientific, St. Leon-Rot, Germany). Results from bioluminescence measurements of variant R1+2 are additionally shown as inlet on a different scale to reflect the low signal intensity.

The results above demonstrate that the *cnfY* RNAT mediates translational thermoregulation to various reporter genes under control of the arabinose-inducible P_*BAD*_ promoter (Figs 1 to 4). In a final set of reporter gene fusion experiments, we measured bioluminescence of transcriptional and translational *luxCDABE* fusions downstream of the native *cnfY* promoter in plasmids pJNS02 [20] or pBO6500), respectively (Fig 5A). The reporter gene fusions include the promoter region as well as 6 nt (transcriptional fusion) or 30 nt (translational fusion) of the *cnfY* coding region. The established *lux* reporter gene system allows monitoring of gene expression in living cells by measuring bioluminescence [37]. Increased transcription at 37°C is consistent (Fig 5B) with previous results [29]. Two results suggest an important contribution of the RNAT to overall *cnfY* expression. First, lower bioluminescence at 25°C in the translational fusion supports an inhibitory function of the secondary RNA structure at low temperatures even when transcript is present. Second, higher temperature-dependent induction of the translational fusion is consistent with translational induction at host body temperature.

We also used translational *luxCDABE* fusions to confirm that the repressive RNAT mutations R1 and R1+2 affect translation of *cnfY* expressed from its natural promoter. This will later become relevant in the infection model (see Fig 8). We grew *Y*. *pseudotuberculosis* cells harboring the translational fusion plasmid with the wildtype RNAT at 25°C and 37°C and measured substantially higher bioluminescence at the higher temperature with the wild type RNAT (Fig 5C). Bioluminescence was strongly reduced in cells harboring the R1 variant and several orders of magnitudes lower in cells carrying the R1+2 variant consistent with the notion that translation is blocked due to the stable RNAT structure. All data described above demonstrate that the *cnfY* thermometer is functional in foreign and native genetic contexts and that it is a major determinant of thermoregulation of *cnfY* expression.

### The *cnfY* RNAT melts gradually at elevated temperatures

**Fig 6:**
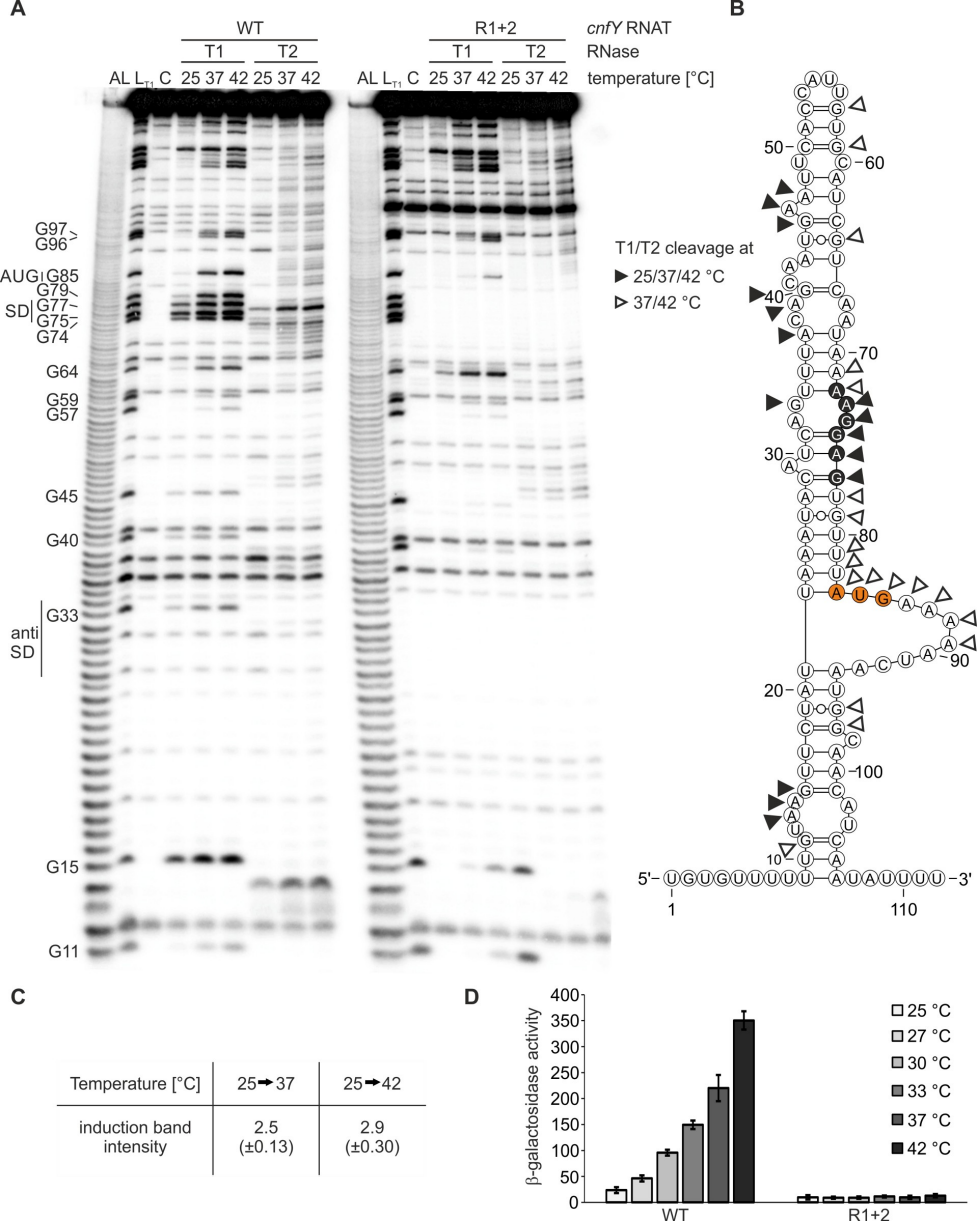
The *cnfY* RNAT undergoes a structural conversion in response to temperature. (A) Enzymatic structure probing of the *cnfY* RNAT (wild type (WT, pBO4465)) and R1+2 variant (pBO4466)) at 25, 37 and 42°C. *In vitro* transcribed RNA was radiolabeled prior to enzymatic digestion with RNase T1 (0.0025 U) and RNase T2 (0.056 U) at the indicated temperatures. Lane L: alkaline ladder, lane L_T1_: RNase T1 cleavage in sequence buffer at 37°C, lane C: RNA treated with water instead of RNase served as control. (B) PARS-derived secondary structure of the *cnfY* RNAT [18] at 25°C. Nucleotides cleaved by RNase T1/T2 at all three temperatures or exclusively at 37 and 42°C are indicated. Nucleotides of the SD region and the AUG start codon are colored in black and orange, respectively. (C) Cleavage signals of the SD sequence (nucleotides G74, G75, and G77 cleaved by RNase T1) were quantified by integrated density quantification using AlphaEaseFC software. The mean density ratio (37°C/25°C) and its standard deviation was calculated from three independent experiments (biological replicate). (D) The wild type (WT; pBO4481) and R1+2 *cnfY* RNAT (pBO6507) were translationally fused to *bgaB* and reporter gene activity was measured as follows. *E*. *coli* DH5α cells harboring the corresponding plasmid (technical triplicate per each construct) were grown to an OD_600_ = 0.5 at 25°C. Afterwards, transcription was induced with 0.01% (w/v) L-arabinose and the culture was split. One sixth remained at 25°C while the other sixths were transferred into pre-warmed flasks at the indicated temperatures. After 30 min of incubation, samples were taken for subsequent β-galactosidase assay. The displayed results represent the mean activities from three independent experiments. Mean standard deviations are indicated as error bars.

To address whether the secondary structure of the *cnfY* RNAT melts open with increasing temperature, we performed enzymatic structure probing on *in vitro* transcribed radiolabeled RNA. The probing experiments were carried out at 25, 37 and 42°C using RNase T1 and RNase T2, which preferentially cleave 3’ of unpaired guanines and unpaired adenines, respectively. Due to these properties both RNases are ideal to monitor unfolding of the purine-rich SD region. The cleavage products were separated on a denaturing 8% polyacrylamide gel (Fig 6A). Noticeable cleavage by RNase T1 or RNase T2 at 25°C suggests that nucleotides 73 to 77 of the SD region are in a partially single-stranded conformation. This is in good agreement with the PARS data-derived secondary structure of the *cnfY* RNAT, where A73 and G74 are located in an internal loop (Fig 6B). Notably, the SD sequence is temperature-sensitive as the cleavage intensities increased from 25°C to 37/42°C (Fig 6C) and nucleotides 71–72 and 78–79 flanking the SD sequence were only accessible at 37 and 42°C. Partially deviant from the PARS-derived secondary structure are the AUG start codon and the subsequent adenosine residues, which are poorly accessible at 25°C. This result suggests tertiary interactions at low temperature that are released at 37 and 42°C.

The cleavage pattern of RNase T1 and T2 in the hairpin up- and downstream of the translation initiation region largely reflects the PARS-constrained secondary structure (Fig 6B). Nucleotides 14 and 15, which are part of or in close proximity to an interior loop, are accessible to RNase T2 at 25°C. The base of the entire hairpin structure is thermo-labile since guanines at position 11, 96, and 97 are solely accessible to RNase T1 at 37 and 42°C. The same is true for the top of the structure, which is in a rather loose conformation since nucleotides 40, 45, 46, and 47 are already accessible to RNases T1 and T2 at 25°C. Guanines 57, 59, and 64, however, are responsible for structure formation at 25°C and become prone to nucleolytic attack only at 37°C. Overall, the structure probing results show that multiple irregularities (bulged residues, internal loops) throughout the needle-like structure of the *cnfY* 5’-UTR contribute to its temperature-sensitive nature, which allows opening after a rather mild transition from environmental to host body temperature. Fully consistent with impaired translation in reporter gene assays, probing of the R1+2 variant (ΔA29, AG32-33CT) revealed a structure that is overall similar to the wild-type structure, but in which the RBS is completely heat-resistant and inaccessible to RNases T1 and T2 at 37 and 42°C (Fig 6A).

To assess whether melting of the RNAT *in vivo* results in a gradual zipper-like or a sudden switch-like response, we assayed β-galactosidase activity of the *bgaB* fusion along a temperature range from 25 to 42°C. The wild-type *cnfY* thermometer induced a gradual increase of reporter gene activity (Fig 6D), indicating zipper-like opening of the RNAT structure. In line with the structure probing results (Fig 6A), the R1+2 fusion was completely unresponsive even at 42°C. These results provide evidence that the RNAT adopts a labile secondary structure at moderate environmental temperatures that reduces accessibility to the RBS to prevent *cnfY* translation when it is not necessary.

### Ribosome binding to the SD region of the *cnfY* RNAT is temperature-controlled

**Fig 7:**
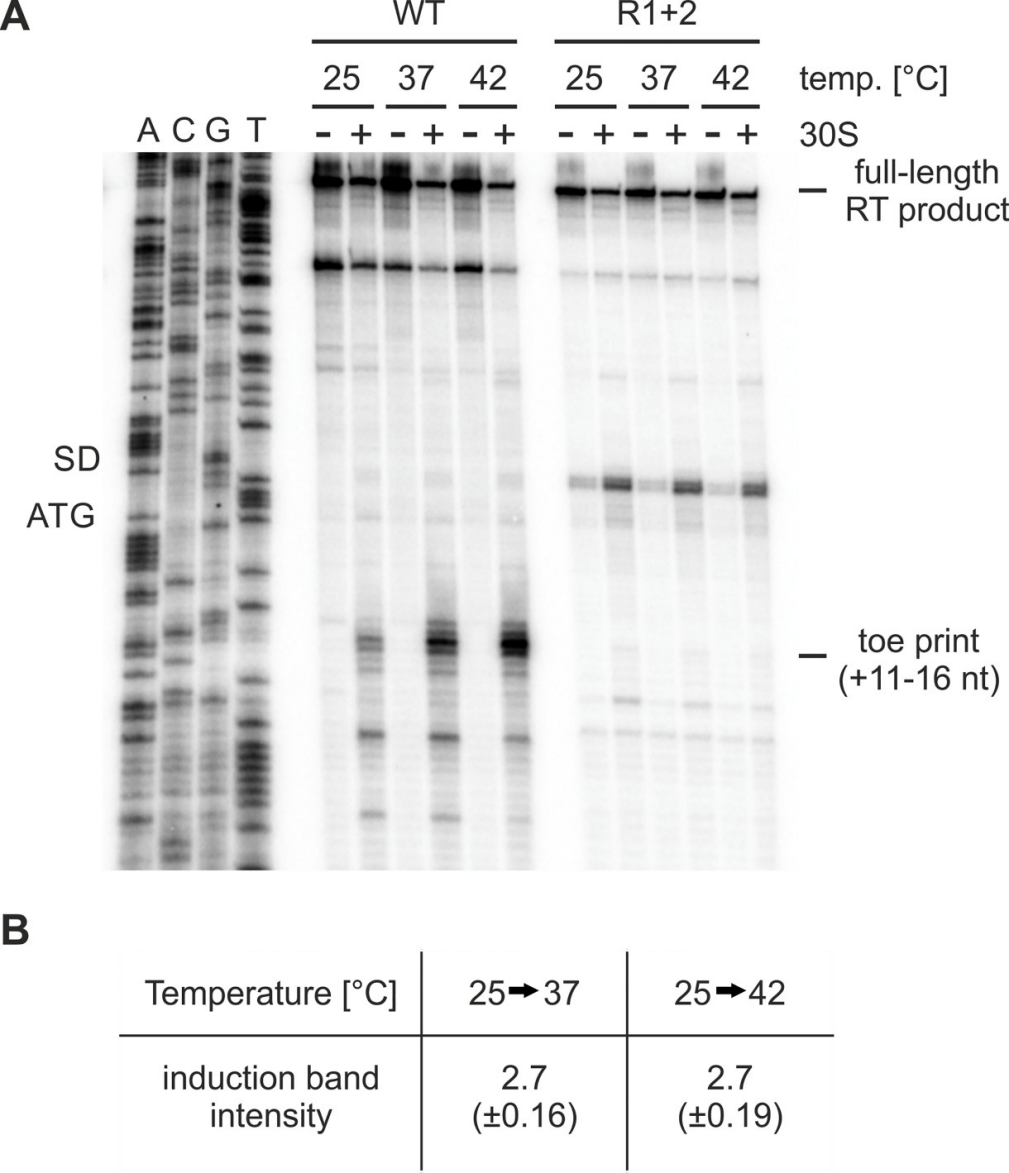
Temperature-controlled ribosome binding to the *cnfY* RNAT. (A) Primer extension inhibition of the wild type (pBO4465) and R1+2 *cnfY* RNAT (pBO4466) in presence (+) or absence (−) of 30S ribosomal subunits was conducted as described in material and methods. The resulting signals correspond to the full-length reverse transcription (RT) product and the termination product (toe print; corresponding to nucleotides +11 to +16 from AUG). ACGT indicate the corresponding DNA sequencing reactions. The SD sequence as well as the ATG codon are labelled. The experiment was performed at least in triplicate (three biological replicates). (B) Toe printing signals (nucleotides +11 to +16 from AUG codon) were quantified by integrated density quantification using AlphaEaseFC software. The mean density ratio (25°C/37°C) and its standard deviation was calculated from three independent experiments.

The mode of action of most RNATs relies on temperature-dependent ribosome binding to the SD sequence, which results from a zipper-like melting process [5]. To address whether the *cnfY* leader sequence follows this regulatory principle we performed toeprinting experiments [34]. After annealing of a *cnfY-*specific primer to the *cnfY* RNAT including 80 nt of the coding region, the RNA was incubated with tRNA^fMet^ and 30S ribosomal subunits at 25, 37 and 42°C. Following primer extension, the reverse transcripts were separated on an 8% denaturing polyacrylamide gel. Toeprinting signals caused by ribosomes acting as a roadblock for reverse transcription appeared at +11 to +16 from the translation start codon only in presence of 30S ribosomal subunits (Fig 7A). Although residual ternary complex formation between mRNA, tRNA^fMet^ and ribosome occurred at 25°C, this process was increased almost 3-fold at elevated temperatures (Fig 7B). Consistent with the absence of RNA melting *in vitro* and translation initiation *in vivo*, no toeprinting signal was detected with the R1+2 variant (ΔA29, AG32-33CT) of the *cnfY* thermometer. An additional signal between the AUG codon and SD sequence appeared, which increased in the presence of 30S ribosomal subunits. Such cDNA products are commonly observed with repressed RNAT structures, for example of the *Salmonella enterica groES* [38] and the *Y*. *pseudotuberculosis lcrF* RNATs [15]. They might result from inhibition or drop-off of the reverse transcriptase by the stable secondary structure. How the 30S subunit favors such premature reverse transcription products is unclear. Altogether, the results strongly suggest a temperature-controlled formation of a ternary complex in absence of any additional bacterial factors.

### The *cnfY* RNAT controls provision of the CNF_Y_ toxin and is critical for virulence

**Fig 8:**
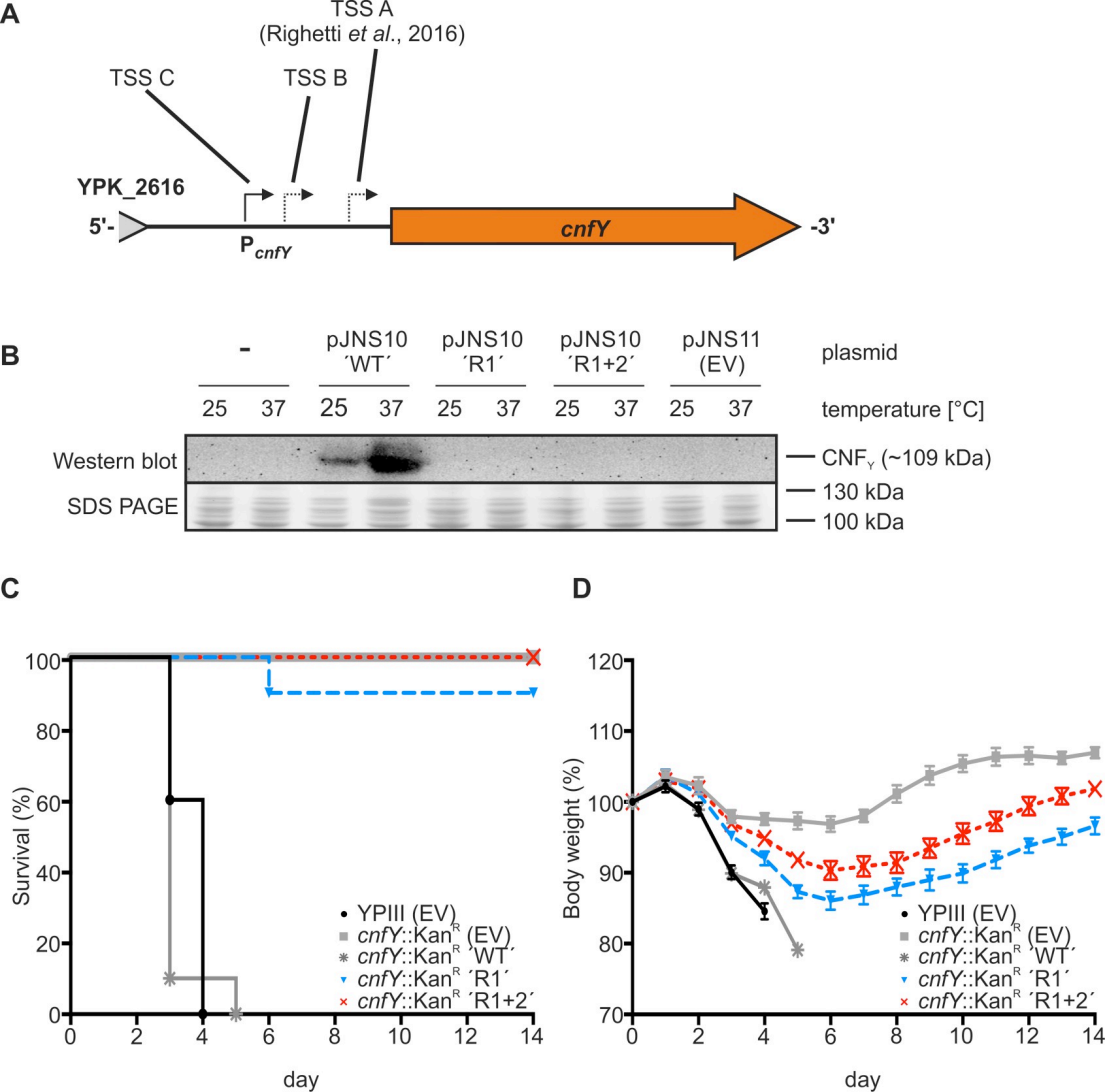
Stabilizing point mutations within the *cnfY* RNAT affect virulence of *Y*. *pseudotuberculosis*. (A) Schematic representation of the *cnfY* promoter region (P_*cnfY*_) and the coding region which are part of the pJNS10 complementation vector [20]. Transcriptional start sites (TSS) identified in a previous study [18] and in this study (see Fig 2) are marked by arrows. (B) *Y*. *pseudotuberculosis* Δ*cnfY* (YP216) cells without vector or harboring vector pJNS10 (*cnfY* complementation vector; [20]), pJNS10 R1 (*cnfY* complementation harboring mutation R1; pBO6503), pJNS10 R1+2 (*cnfY* complementation vector harboring mutation R1+2; pBO6504), and pJNS11 (empty vector) were grown to exponential growth phase (OD_600_ = 0.5). Afterwards, samples were taken for SDS-PAGE as well as Western blot analysis with an antibody against CNF_Y_. The experiment was performed at least in triplicate. (C) Survival and (D) body weight of BALB/c mice (n = 10/strain) was monitored for up to 14 days after oral infection via food pellets with 2x10^9^ CFU of either *Y*. *pseudotuberculosis* YPIII harboring the empty vector pJNS11 (black line + black dots) or the *cnfY* mutant YP147 (*cnfY*::*Kan*^R^) harboring the empty vector pJNS11 (grey line + grey squares) or the *cnfY* complementation vector pJNS10 (pP_*cnfY*_:*cnfY*^+^) including either the wild type (grey line + grey asterisk), the R1 (blue line + blue triangles), or the R1+2 (red line + red crosses) variant of the *cnfY* RNAT. Displayed data represent results from two individual experiments (biological replicates).

A recent study demonstrated the pivotal role of the CNF_Y_ toxin in *Y*. *pseudotuberculosis* pathogenesis [20]. A deletion of *cnfY* rendered *Y*. *pseudotuberculosis* avirulent and this phenotype was restored in presence of the *cnfY* complementation vector pJNS10. This plasmid harbors the complete *cnfY* promoter region followed by the full-length *cnfY* open reading frame (Fig 8A). We inserted the repressive mutations R1 and R1+2 into pJNS10 (pJNS10-R1 and pJNS10-R1+2) and investigated their influence on CNF_Y_ synthesis via immunodetection. *Y*. *pseudotuberculosis* Δ*cnfY* (YP216) cells harboring the respective complementation vectors were grown to exponential growth phase at 25°C and 37°C, and samples were taken for Western blot analysis using a CNF_Y_-specific antibody. Both, the R1 as well as the R1+2 mutation prevented *cnfY* translation at 37°C as no CNF_Y_ was detectable (Fig 8B).

Next, we investigated the relevance of the RNAT in pathogenesis by measuring the survival of BALB/c mice (n = 10 per strain) orally infected with the *Y*. *pseudotuberculosis* wild type carrying the empty vector pJNS11 or a *cnfY-*deficient strain (*cnfY*::Kan^R^, YP147) harboring either pJNS11 or the *cnfY*-complementation vector (pJNS10) with the wild type, the R1, or R1+2 RNAT variant. The survival rate of infected mice was monitored for two weeks (Fig 8C). Mice infected with the wild type strain showed clear signs of infection (e.g. lethargy and rough fur) and reached humane endpoints (cut-off criteria based on health scores). Consistent with previous results [20], infection with the *cnfY* deletion strain did not result in the death of any mice. In presence of the *cnfY* complementation vector, however, the *cnfY* mutant was as virulent as the wild type. Mice infected with *cnfY* deficient mutants harboring pJNS10-R1 exhibited a survival rate of 80% post infection indicating that stabilization of the *cnfY* RNAT reduces virulence potential. The *cnfY*::Kan^R^ strain with the R1+2 RNAT was avirulent like the *cnfY* deletion mutant showing that proper temperature measurement is indispensable for host infection.

Bodyweight measurements of infected mice essentially reflected the survival results. The weight of mice infected with the wild type strain or the *cnfY* mutant complemented with plasmid-borne *cnfY* severely declined after two days of infection (Fig 8D). Mice infected with the *cnfY*::Kan^R^ mutant carrying the empty vector lost little weight and regained it within a week. Mice infected with *Y*. *pseudotuberculosis* mutants carrying stabilized *cnfY* RNATs showed intermediate phenotypes. They lost weight in the first six days but recovered and regained it thereafter. Consistent with the obtained survival rates, the strain carrying the most stable R1+2 RNAT variant least affected the fitness of the mice.

**Fig 9:**
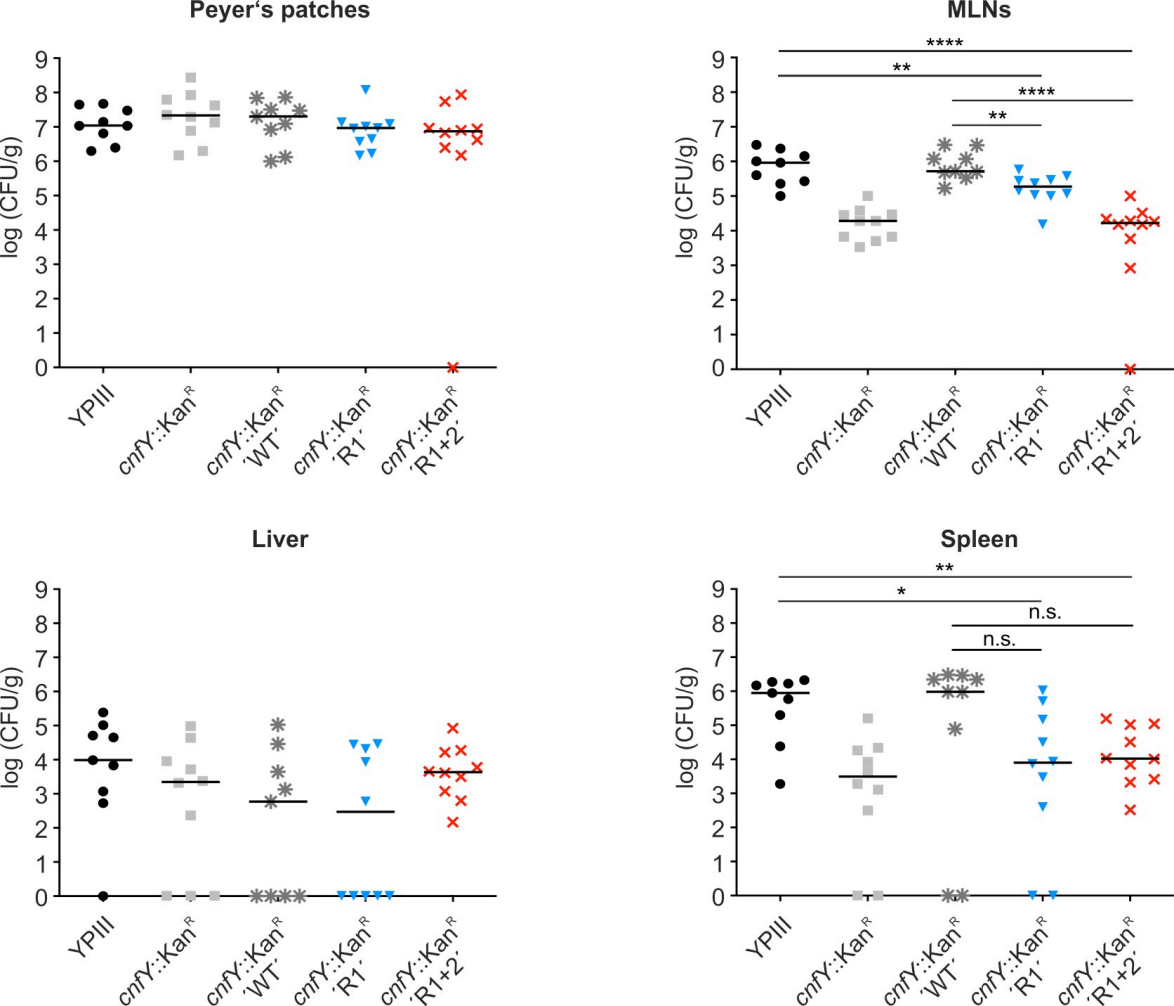
Stabilizing point mutations in the *cnfY* RNAT affects tissue colonization by *Y*. *pseudotuberculosis*. (A) BALB/c mice (n = 10/strain) were orally infected via food pellets with 2x10^8^ CFU of either *Y*. *pseudotuberculosis* YPIII harboring the empty vector pJNS11 (black line + black dots) or the *cnfY* deficient mutant YP147 (*cnfY*::*Kan*^*R*^) harboring the empty vector pJNS11 (grey line + grey squares) or the *cnfY* complementation vector pJNS10 (pP_*cnfY*_:*cnfY*^+^) including the wild type (grey line + grey asterisk), the R1 (blue line + blue triangles; pBO6503)), or the R1+2 (red line + red crosses; pBO6504) variant of the *cnfY* RNAT. 5 days post infection, mice were sacrificed and the number of bacteria in homogenized organs (Peyer‘s patches, mesenteric lymph nodes (MLNs), Liver, and Spleen) was determined by plating. The scatter plots represent data of two independent experiments (5 mice/group). Numbers of CFU per gram were determined by counting viable bacteria on plates. The statistical significances between the wild type and the YP147 mutant were determined by the Mann-Whitney U test. P-values: *: <0.05; **: <0.01, ****: <0.0001. Displayed data represent results from two individual experiments (biological replicates).

Finally, we tested whether stabilization of the *cnfY* thermosensor affects colonization of *Y*. *pseudotuberculosis* within Peyer’s patches, mesenteric lymph nodes (MLNs), liver, or spleen. Mice (n = 10 per strain) were orally infected with 2x10^8^ cells of *Y*. *pseudotuberculosis* YPIII carrying the empty vector or with the *cnfY* deletion mutant carrying *cnfY* complementation vectors with the wild type, R1 or R1+2 RNAT. The number of bacteria in infected organs was determined by plating of organ homogenates five days post infection (Fig 9). No difference in colonization of the Peyer’s patches and the liver were observed between the five *Y*. *pseudotuberculosis* strains. In contrast, significantly reduced numbers of bacteria were recovered from MLNs and spleen when mice were infected with the *cnfY* mutant carrying either an empty vector or the complementation plasmid with the stabilized R1 or R1+R2 thermometers. The latter strain showed the same poor recovery from infected spleen, liver, and MLNs as observed for the *cnfY* deletion mutant. Taken together, the results clearly demonstrate that RNAT-controlled synthesis of CNF_Y_ is crucial for the virulence of *Y*. *pseudotuberculosis*.

## Discussion

Precise spatio-temporal control of virulence gene expression determines the outcome of bacterial infections. Temperature is one of the consistent parameters that correlate with the stage of invasion during the infection of a warm-blooded host [1]. In contrast to transcriptional control mechanisms that proceed through several levels of gene expression, RNATs are one step ahead and directly feed the temperature signal into already existing transcripts and modulate the efficiency of translation initiation [7,8]. Accordingly, several pathogens control expression of their master virulence regulators by RNATs, such as PrfA, ToxT and LcrF in *Listeria monocytogenes*, *Vibrio cholerae* and *Yersinia* species, respectively [15,16,39,40]. Thereby, entire virulence regulons are recruited under temperature control. In addition, individual genes responsible for specific virulence traits can be under direct control of RNATs. Examples are the capsule biosynthesis gene *cssA* in *Neisseria meningitidis* [41] or the heme uptake genes *shuA* and *shuT* in *Shigella dysenteriae* [42,43].

Here we report a detailed structure-function analysis of an RNAT controlling synthesis of the secreted toxin CNF_Y_ in *Y*. *pseudotuberculosis*, which we had identified in a global RNA structurome study [18]. The approx. 100 nt long RNA structure, which reaches more than 20 nt into the coding region contains several remarkable features. Three bulged residues, a two-residue bulge, a large bulge of 10 nt and four short internal loops (Fig 6B) contribute to an overall labile structure ideally suited to calibrate translation in the physiological temperature range like a rheostat. These multiple irregularities in the hairpin structure might provide breathing points also allowing for the observed residual translation even at low temperatures. As one might expect from the many unpaired nucleotides throughout the *cnfY* hairpin, the entire structure is engaged in the melting process. The overall architecture of the *cnfY* RNAT is unique and does not conform to known classes of RNA-based thermosensors, such as the fourU or ROSE elements [5]. The partially paired translation initiation region within an extensive hairpin structure is reminiscent of the *prfA* RNA thermosensor from *L*. *monocytogenes* [39]. However, in contrast to the sudden switch-like response around 37°C in the *prfA* thermometer [41], the temperature-induced opening of the *cnfY* thermosensor was gradual as in many other RNATs.

Another interesting observation relates to the genomic duplication of and polymorphisms in the *cnfY* thermometer, which is remarkably conserved among various *Y*. *pseudotuberculosis* strains and *Y*. *pestis*. Owing to a potential transposase gene (YPK_2616) upstream of *cnfY*, it was postulated that the *cnfY* gene was acquired via horizontal gene transfer [23]. During reevaluation of the transcription start site(s) of the *cnfY* gene, we discovered a major start site upstream of the previously annotated start site. The resulting alternative transcript (TSS C, Fig 2A) contains a duplicated RNAT sequence and is equally functional as the *cnfY* RNAT when present in single copy. A common SNP (U26C) that slightly increases the thermodynamic stability of the secondary structure is common in the upstream copy. The presence of this substitution in the downstream copies in other *Y*. *pseudotuberculosis* strains (IP32593 or PB1/+) and in *Y*. *pestis* did not abrogate thermoregulation (Fig 2C) suggesting an evolutionary pressure to maintain the functionality of the *cnfY* RNAT. Despite having functional thermosensors, several *Y*. *pseudotuberculosis* strains and *Y*. *pestis* do not produce CNF_Y_ because they encode a truncated *cnfY* pseudogene encoding a non-functional toxin [23]. Our results suggest that the ancestral and still intact *cnfY* genes in these organisms have been under temperature control.

The inspection of the 5’-UTRs of a variety of genes coding for CNF family toxins in bacteria other than *Yersinia* suggests that RNAT-mediated thermoregulation of GTPase-activating toxins is quite common. Although the nucleotide sequences upstream of *cnf1* from UPEC, *dnt* from *Bordetella pertussis*, and *vopC* from *Vibrio paraheamolyticus*, which code for the cytotoxic necrotizing factor 1 (CNF1), the dermonecrotizing toxin (DNT), and the CNF-like toxin VopC, respectively [44–46], do not exhibit any sequence conservation among each other or to *Y*. *pseudotuberculosis cnfY* (S3A Fig), all of them are predicted to fold into *cnfY* RNAT-like secondary structures (S3B Fig). Partially or completely occluded SD sequences and/or start codons in extensive stem-loop structures strongly suggest temperature-driven control of translation initiation in all these pathogens.

**Fig 10:**
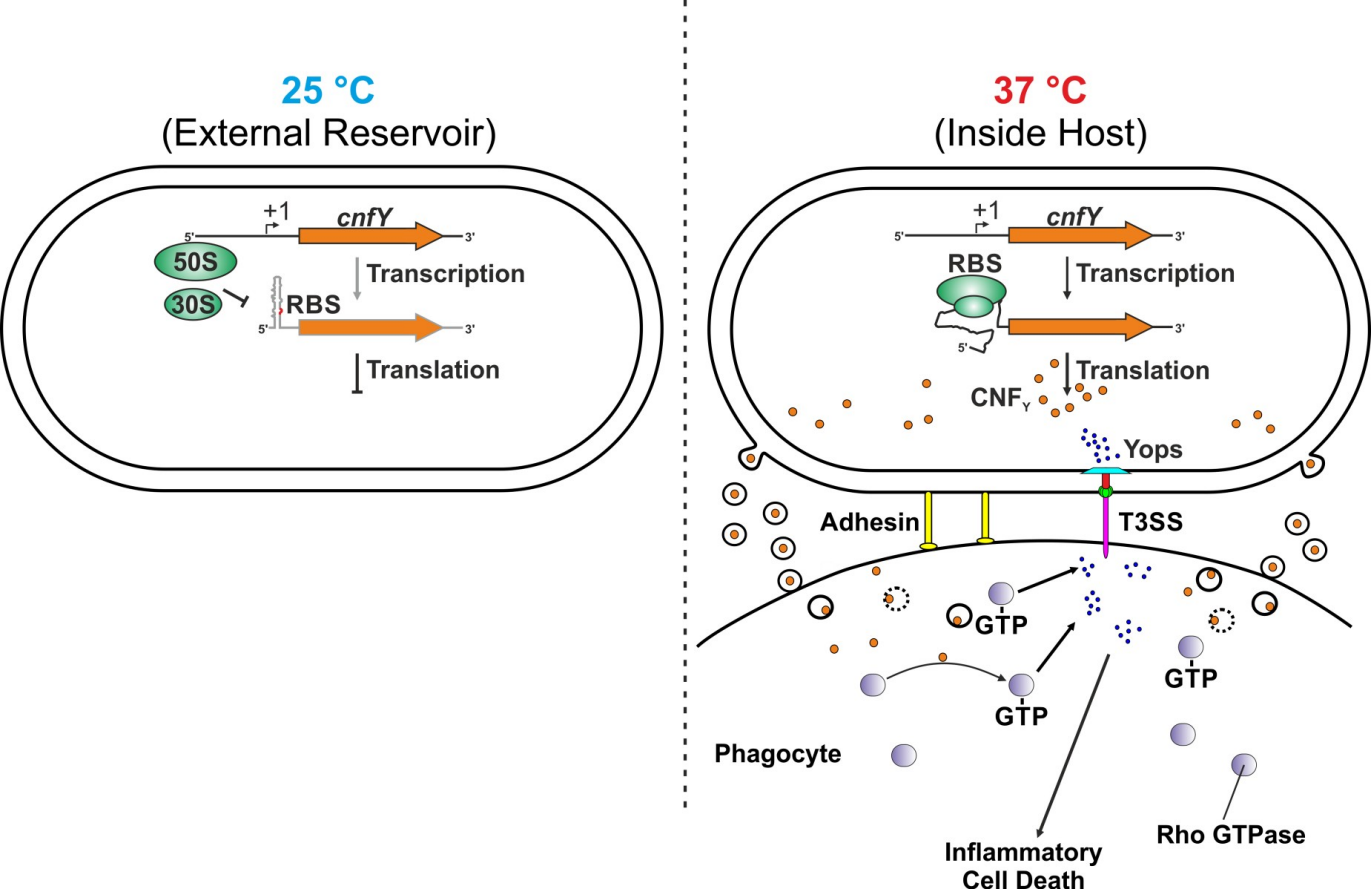
Model of two-layered *cnfY* thermoregulation in *Y*. *pseudotuberculosis*. At moderate temperatures (e.g. 25°C), *cnfY* is weakly transcribed (S4 Table) and translation is hindered by a stem-loop structure in the 5’-UTR of *cnfY*. Within the host (37°C), transcription is enhanced and the RNAT structure melts open, allowing translation of the *cnfY* transcript. During the infection process, the CNF_Y_ toxin is secreted via outer membrane vesicles to the target host cells. Upon reaching the host target cell, the toxin is taken up by endocytosis and released into the cytosol due to a low pH-dependent mechanism. CNF_Y_ then activates small Rho GTPases (RhoA, Rac1, and Cdc42) leading to increased pore formation of the T3SS and boosts Yop effector protein delivery into target cells. Additionally, caspase-1 is activated and triggers secretion of cytokines resulting in cell death due to inflammatory pyroptosis [20,21].

The combined results from this and previous studies (data from transcriptome studies summarized in S4 Table) show that thermoregulation of *cnfY* is a two-layered process comprised of transcriptional and translational control (Fig 10). Notably, even transcription of the *cnfY* pseudogene (YPTB1468) in *Y*. *pseudotuberculosis* IP32953 is temperature-dependent [47]. It is currently unknown how *cnfY* transcription is regulated. It could possibly be due to a temperature-sensitive regulator or the temperature-regulated synthesis of a transcription factor. Transcriptional profiling of *Y*. *pseudotuberculosis* YPIII at 25°C and 37°C suggested an involvement of the cAMP receptor protein (CRP) because temperature-dependent transcription of *cnfY* was lost in a Δ*crp* mutant [29]. The CRP protein is a global transcriptional regulator that, in interplay with the signaling nucleotide cAMP, steers expression of genes associated with sugar utilization [48,49]. Moreover, this regulator was shown to be crucial for virulence of pathogenic *Yersinia* species [50–52], but also other enteropathogens such as UPEC strains [53], *Klebsiella pneumonia* [54,55], *Proteus mirabilis* [56], or *Salmonella enterica* [57].

Although temperature-regulated transcription plays a role in the provision of the CNF_Y_ toxin, our study shows that translational control by the *cnfY* thermometer accounts for a large proportion in this concerted process. Thermoregulation was maintained when transcription was driven from a foreign arabinose-inducible promoter suggesting that pre-existing *cnfY* transcripts are only efficiently translated when body temperature has been reached. Additional upregulation of transcription probably helps boost the toxin levels inside the host and to integrate other host-specific signals than temperature. A complex interplay between transcriptional and translational temperature control of gene expression has also been reported for synthesis of the virulence regulator LcrF from *Y*. *pseudotuberculosis*. Thermal induction involves temperature-regulated proteolysis of the YmoA transcription factor, which represses transcription of the *yscW*-*lcrF* operon at low temperatures, and an intergenic RNAT upstream of *lcrF* [15]. A most recent study demonstrated that the RNA-binding protein CsrA is involved in cell-contact mediated expression of *lcrF* [58]. CsrA binds to two sites within the *lcrF* transcript, one within and one downstream of the SD sequence, which destabilizes the RNAT structure and overcomes translational repression at low temperature (25°C). A dual layer of transcriptional and translational control is also quite common in heat shock gene regulation. Here, many heat-induced genes are under transcriptional control, for example by the alternative heat shock sigma factor σ^32^, plus RNAT-mediated translational control [27,33,59,60].

The CNF_Y_ toxin is one of the important virulence determinants predominantly expressed during the early stage of infection [61]. In this study, we show how crucial individual nucleotides in the untranslated region of a virulence gene are by applying an *in vivo* infection model. Animal models have previously only been used to demonstrate the importance of RNATs upstream of genes coding for virulence master regulators, namely LcrF and ToxT of *Y*. *pseudotuberculosis* and *V*. *cholerae*, respectively [15,40]. Other studies have been restricted to structure-function analysis and *in vitro* models [41–43,62–65]. Here, we demonstrated that converting the loose RNAT structure into a perfectly matched zipper repressed synthesis of CNF_Y_ within the mouse, which made *Y*. *pseudotuberculosis* avirulent. This result reinforces the notion that translational control by the RNAT is the dominant mechanism determining *cnfY* expression in the warm-blooded host. The CNF_Y_ toxin is supposed to amplify crucial virulence factor functions securing fine-tuning of *Y*. *pseudotuberculosis* pathogenicity [20]. Activity is known to be crucial for virulence as deletion of *cnfY* results in reduced mortality rates of infected mice [20]. Moreover, loss of CNF_Y_ reduces inflammatory responses in infected host tissues and reprograms the pathogen's transcriptional response towards a persistent life style [66]. The finding that stabilization of the *cnfY* thermosensor reduced the pathogen’s potential to colonize MLNs and spleens of infected mice is consistent with the observation that absence of CNF_Y_ provokes a significant increase of infiltrating macrophages, monocytes and natural killer cells in the spleen allowing a rapid killing of the pathogen [20].

Unlike many other *Yersinia* virulence factors, CNF_Y_ is not delivered via the T3SS but secreted via outer membrane vesicles (OMVs) [19,20,67]. The CNF-1 toxin from UPEC strains also takes the OMV route [67–69]. Also unlike many other virulence genes in *Yersinia*, the *cnfY* gene is not under temperature control by LcrF. Instead, the horizontally acquired gene is equipped with its own RNAT in order to produce the toxin once the bacterium is inside the warm-blooded host. We find it intriguing that similar RNAT-like structures are likely to exist upstream of various other toxin genes (see above). This finding supports the emerging view that RNA-mediated signal perception is common in pathogenic bacteria and that such RNA-based sensors might be attractive drug targets [70]. Interestingly, a magnesium-binding site in the *Salmonella* fourU thermometer could be targeted by Hexaammincobalt(III)-chlorid [71] and triptycene-based small molecule binding to a central three-way junction in the *E*. *coli rpoH* thermosensor were able to stabilize the structure [72]. These findings promise that antimicrobial compounds interfering with the functionality of RNA thermometers can be developed.

## Supporting information

S1 Table Bacterial strains. The table includes all bacterial strains used in this study.
(DOCX)

S2 Table Oligonucleotide list. The table includes all oligonucleotides used in this study.
(DOCX)

S3 Table Plasmid list. The table includes all plasmids used in this study.
(DOCX)

S4 Table Transcriptional regulation of *cnfY*. The table includes information about transcriptional regulation of *cnfY* obtained from previous studies.
(DOCX)

S1 Fig Expression of *cnfY* is thermoregulated. (A) Detection of CNF_Y_ protein levels at 25°C and 37°C via Western blot analysis. *Y*. *pseudotuberculosis* YPIII cells were grown in LB medium to exponential (OD_600_ = 0.5) and early stationary growth phase (OD_600_ = 1.5) and samples were taken for SDS-PAGE and Western blot analysis using a CNF_Y_-specific antibody. (B) Representative, original Western blot for CNF_Y_ quantification at 25°C and 37°C used for data analysis displayed in Fig 1A. Samples were taken from a *Y*. *pseudotuberculosis* YPIII culture grown in LB medium to exponential growth phase (OD_600_ = 0.5).
(TIF)

S2 Fig Transcriptomic profiling of *cnfY* and genetic organization of the *cnfY* upstream region. (A) Representation of RNA-seq (37°C) based cDNA reads according to Righetti *et al*., 2016 [6] and Nuss *et al*., 2015 [10] visualized via the Artemis genome browser [12]. Different potential TSSs (for details see Fig 1A) are indicated by arrows. (B) Schematic representation of the *cnfY* upstream region (559 nt) including 30 nt of the *cnfY* coding region and 29 nt of the upstream transposase gene found in *Y*. *pseudotuberculosis* strains YPIII (GenBank accession: CP009792), IP2666 pIB1 (CP032566), IP31758 (CP000720), IP32953 (CP009712), and PB1/+ (CP009780) or in *Y*. *pestis* strains CO92 (CP009973) and Pestoides F (CP00668). The *cnfY* RNAT sequence and its upstream duplication are marked in orange. Sequence insertions are marked in blue, whereas asterisks mark nucleotide exchanges within the RNAT sequences (relative to *Y*. *pseudotuberculosis* YPIII). Broken lines indicate sequence deletions relative to the *Y*. *pseudotuberculosis* YPIII genome. Overall sequence identities (relative to *Y*. *pseudotuberculosis* YPIII) are displayed under each species name.
(TIF)

S3 Fig Sequence and structure conservation of leader regions upstream of CNF encoding genes. (A) Multiple alignment of sequences located upstream of genes coding for CNFs or related toxins. Displayed is the alignment of sequences upstream of *cnfY* from *Y*. *pseudotuberculosis* YPIII, *cnf1* from *E*. *coli* O18:K1:H7 UTI89, *dnt* from *Bordetella pertussis* J262, and *vopC* from *Vibrio parahaemolyticus* BB22OP. The multiple sequence alignment was calculated with Clustal Omega (https://www.ebi.ac.uk/Tools/msa/clustalo/) and visualized via jalview application [13]. (B) Secondary structures of the *cnfY* RNAT (-82 nt; [6]) from *Y*. *pseudotuberculosis* YPIII (ΔG° = -13.19; [ΔG°] = kcal*mol^-1^) and the upstream regions (-100 nt and +30 nt from AUG) of *cnf1* from *E*. *coli* O18:K1:H7 UTI89 (ΔG° = -28.20), *dnt* from *B*. *pertussis* J262 (ΔG° = -61.93) and *vopC* from *V*. *parahaemolyticus* BB22OP (ΔG° = -23.05) are displayed. Structure of the *cnfY* RNAT originates from [6]. The remaining structures were predicted via RNAfold [14] with temperature set to 25°C. The proposed SD sequences and AUG start codon are depicted in black and orange, respectively.
(TIF)

S1 References References for supporting information. (DOCX)

## References

[1] Steinmann R, Dersch P. Thermosensing to adjust bacterial virulence in a fluctuating environment. Front Microbiol. 2013; 8(1):85–105. 10.2217/fmb.12.129 23252495

[2] Lam O, Wheeler J, Tang CM. Thermal control of virulence factors in bacteria: A hot topic. Virulence. 2014; 5(8):852–62. 10.4161/21505594.2014.970949 25494856 PMC4601195

[3] Klinkert B, Narberhaus F. Microbial thermosensors. Cell Mol Life Sci. 2009; 66(16):2661–76. 10.1007/s00018-009-0041-3 19554260 PMC11115684

[4] Saita EA, De Mendoza D. Thermosensing via transmembrane protein-lipid interactions. Biochim Biophys Acta. 2015; 1848(9):1757–64. 10.1016/j.bbamem.2015.04.005 25906947

[5] Kortmann J, Narberhaus F. Bacterial RNA thermometers: molecular zippers and switches. Nat Rev Microbiol. 2012; 10(4):255–65. 10.1038/nrmicro2730 22421878

[6] Krajewski SS, Narberhaus F. Temperature-driven differential gene expression by RNA thermosensors. Biochim Biophys Acta. 2014; 1839(10):978–88. 10.1016/j.bbagrm.2014.03.006 24657524

[7] Grosso-Becera MV, Servin-González L, Soberón-Chávez G. RNA structures are involved in the thermoregulation of bacterial virulence-associated traits. Trends Microbiol. 2015; 23(8):509–18. 10.1016/j.tim.2015.04.004 25999019

[8] Loh E, Righetti F, Eichner H, Twittenhoff C, Narberhaus F. RNA thermometers in bacterial pathogens. Microbiol Spectr. 2018; 6(2):RWR-0012-2017 10.1128/microbiolspec.RWR-0012-2017 29623874 PMC11633587

[9] Pechous RD, Sivaraman V, Stasulli NM, Goldman WE. Pneumonic plague: the darker side of *Yersinia pestis*. Trends Microbiol. 2016; 24(3):190–7. 10.1016/j.tim.2015.11.008 26698952

[10] Fredriksson-Ahomaa M. *Yersinia enterocolitica* and *Yersinia pseudotuberculosis*. In: Foodborne Diseases. 2007 p. 79–112 10.1128/9781555815936.ch11

[11] Atkinson S, Williams P. *Yersinia* virulence factors—a sophisticated arsenal for combating host defences. F1000Res. 2016; 5:1370 10.12688/f1000research.8466.1 27347390 PMC4909105

[12] Straley SC, Plano G V., Skrzypek E, Haddix PL, Fields KA. Regulation by Ca^2+^ in the *Yersinia* low-Ca^2+^ response. Mol Microbiol. 1993; 8(6):1005–10. 10.1111/j.1365-2958.1993.tb01644.x 8361348

[13] Straley SC. The low-Ca^2+^ response virulence regulon of human-pathogenic *yersiniae*. Microb Pathog. 1991; 10(2):87–91. 10.1016/0882-4010(91)90069-m 1890954

[14] Straley SC, Perry RD. Environmental modulation of gene expression and pathogenesis in *Yersinia*. Trends Microbiol. 1995; 3(8):310–7. 10.1016/s0966-842x(00)88960-x 8528615

[15] Böhme K, Steinmann R, Kortmann J, Seekircher S, Heroven AK, Berger E, Pisano F, Thiermann T, Wolf-Watz H, Narberhaus F, Dersch P. Concerted actions of a thermo-labile regulator and a unique intergenic RNA thermosensor control *Yersinia* virulence. PLoS Pathog. 2012; 8(2):e1002518 10.1371/journal.ppat.1002518 22359501 PMC3280987

[16] Hoe NP, Goguen JD. Temperature sensing in *Yersinia pestis*: translation of the LcrF activator protein is thermally regulated. J Bacteriol. 1993; 175(24):7901–9. 10.1128/jb.175.24.7901-7909.1993 7504666 PMC206968

[17] Kertesz M, Wan Y, Mazor E, Rinn JL, Nutter RC, Chang HY, Segal E. Genome-wide measurement of RNA secondary structure in yeast. Nature. 2010; 467(7311):103–7. 10.1038/nature09322 20811459 PMC3847670

[18] Righetti F, Nuss AM, Twittenhoff C, Beele S, Urban K, Will S, Bernhart SH, Stadler PF, Dersch P, Narberhaus F. Temperature-responsive *in vitro* RNA structurome of *Yersinia pseudotuberculosis*. Proc Natl Acad Sci USA. 2016; 113(26):7237–42. 10.1073/pnas.1523004113 27298343 PMC4932938

[19] Monnappa AK, Bari W, Seo JK, Mitchell RJ. The cytotoxic necrotizing factor of *Yersinia pseudotuberculosis* (CNFY) is carried on extracellular membrane vesicles to host cells. Sci Rep. 2018; 8(1):14186 10.1038/s41598-018-32530-y 30242257 PMC6155089

[20] Schweer J, Kulkarni D, Kochut A, Pezoldt J, Pisano F, Pils MC, Genth H, Huehn J, Dersch P. The Cytotoxic necrotizing factor of Yersinia pseudotuberculosis (CNF_Y_) enhances inflammation and Yop delivery during infection by activation of Rho GTPases. PLoS Pathog. 2013; 9(11):e1003746 10.1371/journal.ppat.1003746 24244167 PMC3820761

[21] Wolters M, Boyle EC, Lardong K, Trülzsch K, Steffen A, Rottner K, Ruckdeschel K, Aepfelbacher M. Cytotoxic necrotizing factor-Y boosts *Yersinia* effector translocation by activating Rac protein. J Biol Chem. 2013; 288(32):23543–53. 10.1074/jbc.M112.448662 23803609 PMC3949328

[22] Hoffmann C, Pop M, Leemhuis J, Schirmer J, Aktories K, Schmidt G. The *Yersinia pseudotuberculosis* cytotoxic necrotizing factor (CNF_Y_) selectively activates RhoA. J Biol Chem. 2004; 279(16):16026–32. 10.1074/jbc.M313556200 14761941

[23] Lockman HA, Gillespie RA, Baker BD, Shakhnovich E. *Yersinia pseudotuberculosis* produces a cytotoxic necrotizing factor. Infect Immun. 2002; 70(5):2708–14. 10.1128/IAI.70.5.2708-2714.2002 11953417 PMC127951

[24] Knust Z, Schmidt G. Cytotoxic necrotizing factors (CNFs)-a growing toxin family. Toxins (Basel). 2010; 2(1):116–27. 10.3390/toxins2010116 22069550 PMC3206620

[25] Derbise A, Lesic B, Dacheux D, Ghigo JM, Carniel E. A rapid and simple method for inactivating chromosomal genes in *Yersinia*. FEMS Immun Med Microbiol. 2003; 38(2):113–6. 10.1016/S0928-8244(03)00181-0 13129645

[26] Sambrook J, W Russell D. Molecular cloning: a laboratory manual. Cold Spring Harb Lab Press Cold Spring Harb NY 2001; 10.1016/0092-8674(90)90210-6 1205

[27] Gaubig LC, Waldminghaus T, Narberhaus F. Multiple layers of control govern expression of the *Escherichia coli ibpAB* heat-shock operon. Microbiology. 2011; 157(Pt 1):66–76. 10.1099/mic.0.043802-0 20864473

[28] Klinkert B, Cimdins A, Gaubig LC, Roßmanith J, Aschke-Sonnenborn U, Narberhaus F. Thermogenetic tools to monitor temperature-dependent gene expression in bacteria. J Biotechnol. 2012; 160(1–2):55–63. 10.1016/j.jbiotec.2012.01.007 22285954

[29] Nuss AM, Heroven AK, Waldmann B, Reinkensmeier J, Jarek M, Beckstette M, Dersch P. Transcriptomic profiling of *Yersinia pseudotuberculosis* reveals reprogramming of the Crp regulon by temperature and uncovers Crp as a master regulator of small RNAs. PLoS Genet. 2015; 11(3):e1005087 10.1371/journal.pgen.1005087 25816203 PMC4376681

[30] Livak KJ, Schmittgen TD. Analysis of relative gene expression data using real-time quantitative PCR and the 2^-ΔΔCT^ method. Methods. 2001; 25(4):402–8. 10.1006/meth.2001.1262 11846609

[31] Brantl S, Wagner EG. Antisense RNA-mediated transcriptional attenuation occurs faster than stable antisense/target RNA pairing: an *in vitro* study of plasmid pIP501. EMBO J. 1994; 13(15):3599–607. 10.1046/j.1365-2958.2000.01813.x 7520390 PMC395265

[32] Waldminghaus T, Heidrich N, Brantl S, Narberhaus F. FourU: a novel type of RNA thermometer in *Salmonella*. Mol Microbiol. 2007; 65(2):413–24. 10.1111/j.1365-2958.2007.05794.x 17630972

[33] Krajewski SS, Nagel M, Narberhaus F. Short ROSE-like RNA thermometers control IbpA synthesis in *Pseudomonas species*. PLoS One. 2013; 8(5):e65168 10.1371/journal.pone.0065168 23741480 PMC3669281

[34] Hartz D, McPheeters DS, Traut R, Gold L. Extension inhibition analysis of translation initiation complexes. Methods Enzym. 1988; 164:419–25. 10.1016/S0076-6879(88)64058-4 2468068

[35] Roßmanith J, Weskamp M, Narberhaus F. Design of a temperature-responsive transcription terminator. ACS Synth Biol. 2018; 7(2):613–21. 10.1021/acssynbio.7b00356 29191010

[36] Deana A, Belasco JG. Lost in translation: The influence of ribosomes on bacterial mRNA decay. Genes Dev. 2005; 19(21):2526–33. 10.1101/gad.1348805 16264189

[37] Uliczka F, Pisano F, Kochut A, Opitz W, Herbst K, Stolz T, Dersch P. Monitoring of gene expression in bacteria during infections using an adaptable set of bioluminescent, fluorescent and colorigenic fusion vectors. PLoS One. 2011; 6(6):e20425 10.1371/journal.pone.0020425 21673990 PMC3108616

[38] Cimdins A, Roßmanith J, Langklotz S, Bandow JE, Narberhaus F. Differential control of *Salmonella* heat shock operons by structured mRNAs. Mol Microbiol. 2013; 89(4):715–31. 10.1111/mmi.12308 23802546

[39] Johansson J, Mandin P, Renzoni A, Chiaruttini C, Springer M, Cossart P. An RNA thermosensor controls expression of virulence genes in *Listeria monocytogenes*. Cell. 2002; 110(5):551–61. 10.1016/s0092-8674(02)00905-4 12230973

[40] Weber GG, Kortmann J, Narberhaus F, Klose KE. RNA thermometer controls temperature-dependent virulence factor expression in *Vibrio cholerae*. Proc Natl Acad Sci USA. 2014; 111(39):14241–6. 10.1073/pnas.1411570111 25228776 PMC4191814

[41] Loh E, Kugelberg E, Tracy A, Zhang Q, Gollan B, Ewles H, Chalmers R, Pelicic V, Tang CM. Temperature triggers immune evasion by *Neisseria meningitidis*. Nature. 2013; 502(7470):237–40. 10.1038/nature12616 24067614 PMC3836223

[42] Kouse AB, Righetti F, Kortmann J, Narberhaus F, Murphy ER. RNA-Mediated thermoregulation of iron-acquisition genes in *Shigella dysenteriae* and pathogenic *Escherichia coli*. PLoS One. 2013; 8(5) 10.1371/journal.pone.0063781 23704938 PMC3660397

[43] Wei Y, Kouse AB, Murphy ER. Transcriptional and posttranscriptional regulation of *Shigella shuT* in response to host-associated iron availability and temperature. Microbiologyopen. 2017; 6(3):e00442 10.1002/mbo3.442 28127899 PMC5458455

[44] Schmidt G, Sehr P, Wilm M, Mann M, Aktories K. Gln 63 of Rho is deamidated by *Escherichia coli* cytotoxic necrotizing factor-1. Nature. 1997; 387(June):725–9. 10.1038/42735 9192900

[45] Fukui A, Horiguchi Y. *Bordetella* dermonecrotic toxin exerting toxicity through activation of the small GTPase Rho. J Biochem. 2004; 136(4):415–9. 10.1093/jb/mvh155 15625308

[46] Zhang L, Krachler AM, Broberg CA, Li Y, Mirzael H, Gilpin CJ, Orth K. Type III effector VopC mediates invasion for *Vibrio* species. Cell Rep. 2012; 1(5):453–60. 10.1016/j.celrep.2012.04.004 22787576 PMC3392014

[47] Nuss AM, Beckstette M, Pimenova M, Schmühl C, Opitz W, Pisano F, Heroven AK, Dersch P. Tissue dual RNA-seq allows fast discovery of infection-specific functions and riboregulators shaping host–pathogen transcriptomes. Proc Natl Acad Sci USA. 2017; 114(5):E791–800. 10.1073/pnas.1613405114 28096329 PMC5293080

[48] Kolb A, Busby S, Buc H, Garges S, S A. Transcriptional regulation by cAMP and its receptor protein. Annu Rev Biochem. 1993; 62:749–95. 10.1146/annurev.bi.62.070193.003533 8394684

[49] Deutscher J. The mechanisms of carbon catabolite repression in bacteria. Curr Opin Microbiol. 2008; 11(2):87–93. 10.1016/j.mib.2008.02.007 18359269

[50] Lathem WW, Schroeder JA, Bellows LE, Ritzert JT, Koo JT, Price PA, Caulfield AJ, Goldman WE. Posttranscriptional regulation of the *Yersinia pestis* cyclic AMP receptor protein Crp and impact on virulence. MBio. 2014; 5(1):1–12. 10.1128/mbio.01038-13 24520064 PMC3950509

[51] Heroven AK, Sest M, Pisano F, Scheb-Wetzel M, Steinmann R, Böhme K, Klein J, Münch R, Schomburg D, Dersch P. Crp induces switching of the CsrB and CsrC RNAs in *Yersinia pseudotuberculosis* and links nutritional status to virulence. Front Cell Infect Microbiol. 2012; 2(158):1–21. 10.3389/fcimb.2012.00158 23251905 PMC3523269

[52] Zhan L, Han Y, Yang L, Geng J, Li Y, Gao H, Guo Z, Fan W, Li G, Zhang L, Qin C, Zhou D, Yang R. The cyclic AMP receptor protein, CRP, is required for both virulence and expression of the minimal CRP regulon in *Yersinia pestis* biovar microtus. Infect Immun. 2008; 76(11):5028–37. 10.1128/IAI.00370-08 18710863 PMC2573370

[53] Donovan GT, Paul Norton J, Bower JM, Mulvey MA. Adenylate cyclase and the cyclic AMP receptor protein modulate stress resistance and virulence capacity of uropathogenic *Escherichia coli*. Infect Immun. 2013; 81(1):249–58. 10.1128/IAI.00796-12 23115037 PMC3536135

[54] Xue J, Tan B, Yang S, Luo M, Xia H, Zhang X, Zhou X, Yang X, Yang R, Li Y, Qiu J. Influence of cAMP receptor protein (CRP) on bacterial virulence and transcriptional regulation of *allS* by CRP in *Klebsiella pneumoniae*. Gene. 2016; 593(1):28–33. 10.1016/j.gene.2016.08.006 27502416

[55] Ou Q, Fan J, Duan D, Xu L, Wang J, Zhou D, Yang H, Li B. Involvement of cAMP receptor protein in biofilm formation, fimbria production, capsular polysaccharide biosynthesis and lethality in mouse of *Klebsiella pneumoniae* serotype K1 causing pyogenic liver abscess. J Med Microbiol. 2017; 66(1):1–7. 10.1099/jmm.0.000391 27902401

[56] Tsai YL, Chien HF, Huang KT, Lin WY, Liaw SJ. CAMP receptor protein regulates mouse colonization, motility, fimbria-mediated adhesion, and stress tolerance in uropathogenic *Proteus mirabilis*. Sci Rep. 2017; 7(1):1–14. 10.1038/s41598-016-0028-x 28779108 PMC5544767

[57] El Mouali Y, Gaviria-Cantin T, Sánchez-Romero MA, Gibert M, Westermann AJ, Vogel J, Balsalobre C. CRP-cAMP mediates silencing of *Salmonella* virulence at the post-transcriptional level. PLoS Genet. 2018; 14(6):1–26. 10.1371/journal.pgen.1007401 29879120 PMC5991649

[58] Kusmierek M, Hoßmann J, Witte R, Opitz W, Vollmer I, Volk M, Heroven AK, Wolf-Watz H, Dersch P. A bacterial secreted translocator hijacks riboregulators to control type III secretion in response to host cell contact. PLoS Pathog. 2019; 15(6):e1007813 10.1371/journal.ppat.1007813 31173606 PMC6583979

[59] Waldminghaus T, Fippinger A, Alfsmann J, Narberhaus F. RNA thermometers are common in α- and γ-proteobacteria. Biol Chem. 2005; 386(12):1279–86. 10.1515/BC.2005.145 16336122

[60] Krajewski SS, Joswig M, Nagel M, Narberhaus F. A tricistronic heat shock operon is important for stress tolerance of *Pseudomonas putida* and conserved in many environmental bacteria. Env Microbiol. 2014; 16(6):1835–53. 10.1111/1462-2920.12432 24612349

[61] Avican K, Fahlgren A, Huss M, Heroven AK, Beckstette M, Dersch P, Fällman M. Reprogramming of *Yersinia* from virulent to persistent mode revealed by complex *in vivo* RNA-seq analysis. PLoS Pathog. 2015; 11(1):1–28. 10.1371/journal.ppat.1004600 25590628 PMC4295882

[62] Loh E, Lavender H, Tan F, Tracy A, Tang CM. Thermoregulation of meningococcal fHbp, an important virulence factor and vaccine antigen, is mediated by anti-ribosomal binding site sequences in the open reading frame. PLoS Pathog. 2016; 12(8):1–21. 10.1371/journal.ppat.1005794 27560142 PMC4999090

[63] Grosso-Becerra M V., Croda-Garcia G, Merino E, Servin-Gonzalez L, Mojica-Espinosa R, Soberon-Chavez G. Regulation of *Pseudomonas aeruginosa* virulence factors by two novel RNA thermometers. Proc Natl Acad Sci USA. 2014; 111(43):15562–7. 10.1073/pnas.1402536111 25313031 PMC4217398

[64] Matsunaga J, Schlax PJ, Haake DA. Role for *cis*-Acting RNA sequences in the temperature-dependent expression of the multiadhesive Lig proteins in *Leptospira interrogans*. J Bacteriol. 2013; 195(22):5092–101. 10.1128/JB.00663-13 24013626 PMC3811586

[65] Li G-W, Burkhardt D, Gross C, Weissman JS. Quantifying absolute protein synthesis rates reveals principles underlying allocation of cellular resources. Cell. 2014; 157(3):624–35. 10.1016/j.cell.2014.02.033 24766808 PMC4006352

[66] Heine W, Beckstette M, Heroven AK, Thiemann S, Heise U, Nuss AM, Pisano F, Strowig T, Dersch P. Loss of CNF_Y_ toxin-induced inflammation drives *Yersinia pseudotuberculosis* into persistency. PLoS Pathog. 2018; 14(2):1–32. 10.1371/journal.ppat.1006858 29390040 PMC5811047

[67] Blumenthal B, Hoffmann C, Aktories K, Backert S, Schmidt G. The cytotoxic necrotizing factors from *Yersinia pseudotuberculosis* and from *Escherichia coli* bind to different cellular receptors but take the same route to the cytosol. Infect Immun. 2007; 75(7):3344–53. 10.1128/IAI.01937-06 17438028 PMC1932955

[68] Kouokam JC, Wai SN, Fällman M, Hacker J, Uhlin BE, Fa M, Dobrindt U. Active cytotoxic necrotizing factor 1 associated with outer membrane vesicles from uropathogenic *Escherichia coli*. Infect Immun. 2006; 74(4):2022–30. 10.1128/IAI.74.4.2022-2030.2006 16552031 PMC1418910

[69] Davis JM, Carvalho HM, Rasmussen SB, O’Brien AD. Cytotoxic necrotizing factor type 1 delivered by outer membrane vesicles of uropathogenic *Escherichia coli* attenuates polymorphonuclear leukocyte antimicrobial activity and chemotaxis. Infect Immun. 2006; 74(8):4401–8. 10.1128/IAI.00637-06 16861625 PMC1539604

[70] Ignatov D, Johansson J. RNA-mediated signal perception in pathogenic bacteria. Wiley Interdiscip Rev RNA. 2017; 8(6):e1429 10.1002/wrna.1429 28792118

[71] Rinnenthal J, Klinkert B, Narberhaus F, Schwalbe H. Modulation of the stability of the *Salmonella* fourU-type RNA thermometer. Nucleic Acids Res. 2011; 39(18):8258–70. 10.1093/nar/gkr314 21727085 PMC3185406

[72] Barros SA, Yoon I, Chenoweth DM. Modulation of the *E*. *coli rpoH* temperature sensor with triptycene-based small molecules. Angew Chem Int Ed. 2016; 55(29):8258–61. 10.1002/anie.201601626 27240201 PMC5056428

