# Supplementary material for: An RNA thermometer dictates production of a secreted bacterial toxin
Source: PLoS Pathog. 2020 Jan 17;16(1):e1008184. doi: 10.1371/journal.ppat.1008184 (PMC6992388; doi:10.1371/journal.ppat.1008184)
Supplement: S1 Table — The table includes all bacterial strains used in this study. (DOCX) [file ppat.1008184.s001.docx]

**S1 Table. Bacterial strains**

| **Strain** | **Relevant characteristics** | **Reference** |
| --- | --- | --- |
| *Yersinia pseudotuberculosis* YPIII | pIB1, wild type | [1] |
| *Yersinia pseudotuberculosis* YP147 | pIB1, *cnfY*::*Kan^R^* (ΔYPK_2615::*Kan^R^*) | [2] |
| *Yersinia pseudotuberculosis* YP216 | pIB1, Δ*cnfY* (ΔYPK_2615) | J. Schweer |
| *Escherichia coli* DH5α | *supE44*, Δ*lacU169* (ψ80*lacZ*Δ*M15*), *hsdR17*, *recA1*, *gyrA96*, *thi1*, *relA1* | [3] |
