# Supplementary material for: An RNA thermometer dictates production of a secreted bacterial toxin
Source: PLoS Pathog. 2020 Jan 17;16(1):e1008184. doi: 10.1371/journal.ppat.1008184 (PMC6992388; doi:10.1371/journal.ppat.1008184)
Supplement: S2 Table — The table includes all oligonucleotides used in this study. (DOCX) [file ppat.1008184.s002.docx]

**S2 Table. Oligonucleotide list**

| **Name** | **Purpose** | **Plasmid** | **Sequence (5‘->3‘)** |
| --- | --- | --- | --- |
| lcrF_ICR_fw | forward primer to amplify the ICR between pYV0075(*yscW*) and pYV0076(*lcrF*)plus 9 bp of *lcrF* coding region ( 123 to +9 bp from *lcrF* AUG) | pBO4477 | TTGCTAGCGTGATTTATTATATTGGTTTTGG |
| lcrF_ICR_rv | reverse primer to amplify the ICR between pYV0075(*yscW*) and pYV0076(*lcrF*) plus 9 bp of *lcrF* coding region ( 123 to +9 bp from *lcrF* AUG) | pBO4477 | AAGAATTCTGATGCCATAAATGTTATACTG |
| YPK_2615utr_fw | forward primer to amplify the YPK_2615 (*cnfY*) RNAT plus 30 bp of *cnfY* coding region (-82 to +30 bp from *cnfY* ATG) | pBO4478 | TTGCTAGCTGTGTTTTTTGTAAGTTCTATTAAAT |
| YPK_2615utr_rv | reverse primer to amplify the YPK_2615 (*cnfY*) RNAT plus 30 bp of *cnfY* coding region (-82 to +30 bp from *cnfY* ATG) | pBO4478 | TTGAATTCAAAATATTGATGTTGCCATTGATT |
| YPK2615_QC2_fw | mutagenesis forward primer to restore the original sequence of YPK_2615 (*cnfY*) 5‘ UTR | pBO4481 | CATCAGTTTACAGCAATGAATTCACCATTGTGCATCGTCAAT |
| YPK2615_QC2_rv | mutagenesis reverse primer to restore the original sequence of YPK_2615 (*cnfY*) 5‘ UTR | pBO4481 | ATTGACGATGCACAATGGTGAATTCATTGCTGTAAACTGATG |
| cnfY_TSSl_fw | forward primer to amplify the 5’ UTR of YPK_2615 (*cnfY*) plus 30 bp of *cnfY* coding region (-82 to +30 bp from *cnfY* ATG) | pBO6527 | TTGCTAGCTTTCATGTGAGTGATATTCTGTA |
| cnfY_TSSl_rv | reverse primer to amplify the 5’ UTR of YPK_2615 (*cnfY*) plus 30 bp of *cnfY* coding region (-82 to +30 bp from *cnfY* ATG) | pBO6527 | TTGAGCTCAAAATATTGATGTTGCCATTGATT |
| bgaB_Eco_Sac_fw | forward primer for *Eco*RI(GAATTC) -> *Sac*I(GAGCTC) replacement in pBAD2-*bgaB*-His | pBO6524 | CTTAGAAGGAGAAATGACTGAGCTCGTGTTATCCTCAATTTGTTAC |
| bgaB_Eco_Sac_rv | reverse primer for *Eco*RI(GAATTC) -> *Sac*I(GAGCTC) replacement in pBAD2-*bgaB*-His | pBO6524 | GTAACAAATTGAGGATAACACGAGCTCAGTCATTTCTCCTTCTAAG |
| cnfY_Sac_Eco_fw | forward primer for *Sac*I(GAGCTC) -> *Eco*RI(GAATTC) replacement in pBO6527 | pBO6528 | GGCAACATCAATATTTTGAATTCGTGTTATCCTCAATTTGTTAC |
| cnfY_Sac_Eco_rv | reverse primer for *Sac*I(GAGCTC) -> *Eco*RI(GAATTC) replacement in pBO6527 | pBO6528 | GTAACAAATTGAGGATAACACGAATTCAAAATATTGATGTTGCC |
| cnfY_Ype_fw | mutagenesis forward primer to introduce the mutation T26C into YPK_2615 (*cnfY*) 5’‑UTR | pBO6523 | GTTTTTTGTAAGTTCTATTAAACACATCAGTTTACAGCAATG |
| cnfY_Ype_rv | mutagenesis reverse primer to introduce the mutation T26C into YPK_2615 (*cnfY*) 5’‑UTR | pBO6523 | CATTGCTGTAAACTGATGTGTTTAATAGAACTTACAAAAAAC |
| cnfY_R1_fw | mutagenesis forward primer to introduce the mutation AG32‑33CT (R 1) into YPK_2615 (*cnfY*) 5’‑UTR | pBO3190  pBO4481  pBO4465 | TGTAAGTTCTATTAAATACAUCCTTTTACAGCAATGAATTCACC |
| cnfY _ R1_rv | mutagenesis reverse primer to introduce the mutation AG32‑33CT (R 1) into YPK_2615 (*cnfY*) 5’‑UTR | pBO3190  pBO4481  pBO4465 | GGTGAATTCATTGCTGTAAA  AGGUTGTATTTAATAGAACTTACA |
| cnfY _ R2_fw | mutagenesis forward primer to introduce the mutation A29Δ (R2) into YPK_2615 (*cnfY*) 5’‑UTR | pBO3190  pBO4481 | GTAAGTTCTATTAAATACTCAGTTTACAGCAATGAATTCACC |
| cnfY _ R2_rv | mutagenesis reverse primer to introduce the mutation A29Δ (R2) into YPK_2615 (*cnfY*) 5’‑UTR | pBO3190  pBO4481 | GGTGAATTCATTGCTGTAAACTGAGTATTTAATAGAACTTAC |
| cnfY _ R1+2_fw | mutagenesis forward primer to introduce the mutation ATCAG29-33TCCT (R1+2) into YPK_2615 (*cnfY*) 5’‑UTR | pBO3190  pBO4481  pBO4465 | GTAAGTTCTATTAAATACTCCTTTTACAGCAATGAATTCACC |
| cnfY _ R1+2_rv | mutagenesis forward primer to introduce the mutation ATCAG29-33TCCT (R 1+2) into YPK_2615 (*cnfY*) 5’‑UTR | pBO3190  pBO4481  pBO4465 | GGTGAATTCATTGCTGTAAAAGGAGTATTTAATAGAACTTAC |
| cnfY_RO_fw | forward primer to amplify YPK_2615 (*cnfY*) 5’ UTR with a T7 promoter for the construction of the runoff plasmids | pBO4465 | AGAAATTAATACGACTCACTATAGGGTGTGTTTTTTGTAAGTTCTATTAA |
| cnfY_RO_rv | YPK_2615 (*cnfY*) 5’ UTR + 80 bp from ATG; reverse primer with *Eco*RV site for the construction of the runoff plasmid for structure probing and toe printing | pBO4465 | AGATATCACTACTTTTTCTGGGGACGG |
| cnfY_transla_fw | forward primer to amplify the YPK_2615 (*cnfY*) promoter region plus 5’ UTR of *cnfY* plus 30 bp of *cnfY* coding region (-82 to +30 bp from *cnfY* ATG) | pBO6501  pBO6502  pBO6503 | GGGGGGGATCCTATTGACAAACAAAATGAAGCAAGATAG |
| cnfY_transla_rv | Reverse primer to amplify the YPK_2615 (*cnfY*) promoter region plus 5’ UTR of *cnfY* plus 30 bp of *cnfY* coding region (-82 to +30 bp from *cnfY* ATG) | pBO6501  pBO6502  pBO6503 | GGGGTCGACAAAATATTGATGTTGCCATTGATT |
| PcnfY_BamHI_fw | forward primer to amplify the YPK_2615 (*cnfY*) promoter region plus 5’ UTR of *cnfY* plus 30 bp of *cnfY* coding region (-82 to +117 bp from *cnfY* ATG) | pBO6503  p BO6504 | GCGCGAGCTCGGATCCTATTGACAAACAAAATGAAGCAAG |
| cnfY_NcoI_rv | Reverse primer to amplify the YPK_2615 (*cnfY*) promoter region plus 5’ UTR of *cnfY* plus 30 bp of *cnfY* coding region (-82 to +117 bp from *cnfY* ATG) | pBO6503  pBO6504 | TTTCCATGGAAGTGTTGTACTAAAC |
| GmR_NcoI_fw | Forward primer to amplify a Gen^R^ cassette | pBO4499 | CGCGCCATGGTTGACATAAGCCTGTTCGG |
| GmR_BamHI_rv | Reverse primer to amplify a Gen^R^ cassette | pBO4499 | GCGCGGATCCGTTGTGACAATTTACCGAAC |
| GFPprobe_fw | RNA probe for Northern blot analysis | - | CTTCTTCAAGGATGACGGGAACTACA |
| GFPprobe_rv | RNA probe for Northern blot analysis | - | **GAAATTAATACGACTCACTATAGGG**GACAGGGCCATCGCCGATGGGCGT |
| cnfY_qRT_fw | Forward primer to amplify the *cnfY* transcript during qRT-PCR | - | CAGCGAGATTATGCCCTGAATG |
| cnfY_qRT_rv | Reverse primer to amplify the *cnfY* transcript during qRT-PCR | - | GAGCGACGCTCTTCATAAGTATC |
| gyrB_qRT_fw | Forward primer to amplify the *gyrB* transcript during qRT-PCR | - | TCGCCGTGAAGGTAAAGTTC |
| gyrB_qRT_rv | Reverse primer to amplify the *gyrB* transcript during qRT-PCR | - | CGTAATGGAAGTGGTCTTCT |
| nuoB_qRT_fw | Forward primer to amplify the *nuoB* transcript during qRT-PCR | - | GATCCTCTCGAGCAACATG |
| nuoB_qRT_rv | Reverse primer to amplify the *nuoB* transcript during qRT-PCR | - | TAAAGCAGGTTCCGGCCA |
