# Supplementary material for: An RNA thermometer dictates production of a secreted bacterial toxin
Source: PLoS Pathog. 2020 Jan 17;16(1):e1008184. doi: 10.1371/journal.ppat.1008184 (PMC6992388; doi:10.1371/journal.ppat.1008184)
Supplement: S3 Table — The table includes all plasmids used in this study. (DOCX) [file ppat.1008184.s003.docx]

**S3 Table. Plasmid list**

| **Plasmid** | **Relevant characteristics** | **Reference** |
| --- | --- | --- |
| pUC18 | Cloning vector; Ap^r^ | [4] |
| pBAD2-*gfp* | *gfp* reporter gene vector, Ap^r^, Gen^r^, *araC*, P*_BAD_* promoter | [5] |
| pBAD2-*bgaB*-His | *bgaB* reporter gene vector, Ap^r^, *araC*, P*_BAD_* promoter, His‑Tag at the C‑terminal of BgaB | [6] |
| pFU53 | *luxCDABE* reporter gene vector, Ap^r^; pSC101* | [7] |
| pJNS02 | Ap^r^, pSC101*, promoter region of YPK_2615 (*cnfY*) plus 5’ UTR of *cnfY* plus 30 bp of *cnfY* coding region (-82 to +30 bp from ATG of *cnfY*) fused to *luxCDABE* operon, transcriptional fusion | [2] |
| pJNS10 | *cnfY* complementation vector; promoter region of YPK_2615 (*cnfY*) plus 5’ UTR of *cnfY* plus coding region of *cnfY*,Ap^r^, pSC101* | [2] |
| pJNS11 | empty vector (*cnfY* complementation), Ap^r^, pSC101* | [2] |
| pBO3146 | pBAD2-*bgaB*-His; ICR between pYV0075(*yscW*) and pYV0076(*lcrF*) plus 9 bp of *lcrF* coding region ( 123 to +9 bp from *lcrF* ATG) | [6] |
| pBO3190 | pUC18; YPK_2615 (*cnfY*) RNAT plus 30 bp of *cnfY* coding region (-82 to +30 bp from cnfY ATG), mutant TT48,49AA | [6] |
| pBO3192 | pBAD2-*bgaB*-His; YPK_2615 (*cnfY*) RNAT plus 30 bp of *cnfY* coding region (-82 to +30 bp from *cnfY* ATG) | [6] |
| pBO4449 | pBAD2-*bgaB*-His; YPK_2615 (*cnfY*) RNAT plus 30 bp of *cnfY* coding region (-82 to +30 bp from *cnfY* ATG), mutant R1+2 ATCAG29-33TCCT | This study |
| pBO4465 | pUC18; YPK_2615 (*cnfY*) RNAT plus coding region (-82 to +80 bp from *cnfY* ATG); runoff plasmid for structure probing and primer extension inhibition | This study |
| pBO4466 | pUC18; YPK_2615 (*cnfY*) 5’ UTR plus coding region (-82 to +80 bp from *cnfY* ATG), mutant R1+2 ATCAG29-33TCCT; runoff plasmid for structure probing and primer extension inhibition | This study |
| pBO4477 | pBAD2-*gfp*; ICR between pYV0075(*yscW*) and pYV0076(*lcrF*) plus 9 bp of *lcrF* coding region ( 123 to +9 bp from *lcrF* ATG) | This study |
| pBO4478 | pBAD2-*gfp*; YPK_2615 (*cnfY*) RNAT plus 30 bp of *cnfY* coding region (-82 to +30 bp from *cnfY* ATG), mutant TT48,49AA | This study |
| pBO4481 | pBAD2-*gfp*; YPK_2615 (*cnfY*) RNAT plus 30 bp of *cnfY* coding region (-82 to +30 bp from *cnfY* ATG) | This study |
| pBO4493 | pEX-K168 (Eurofins Genomics, Ebersberg, Germany); promoter region of YPK_2615 (*cnfY*) plus 5’ UTR of *cnfY* plus *cnfY* coding region (-82 to +117 bp from *cnfY* ATG), mutant R1 AG32 | This study |
| pBO4494 | pEX-K168 (Eurofins Genomics, Ebersberg, Germany); promoter region of YPK_2615 (*cnfY*) plus 5’ UTR of *cnfY* plus *cnfY* coding region (-82 to +117 bp from *cnfY* ATG), mutant R1+2 ATCAG29-33TCCT | This study |
| pBO4499 | pJNS10; exchange of promoter region of YPK_2615 (*cnfY*) plus 5’ UTR of *cnfY* plus plus *cnfY* coding region (-82 to +117 bp from *cnfY* ATG) in pJNS10 against Gen^R^ cassette | This study |
| pBO4610 | pBAD2-*bgaB*-His; YPK_2615 (*cnfY*) RNAT plus 30 bp of *cnfY* coding region (-82 to +30 bp from *cnfY* ATG), mutant R1 AG32 33CT | [6] |
| pBO4611 | pBAD2-*bgaB*-His; YPK_2615 (*cnfY*) RNAT plus 30 bp of *cnfY* coding region (-82 to +30 bp from *cnfY* ATG), mutant R2 A29Δ | [6] |
| pBO6500 | pFU53; promoter region of YPK_2615 (*cnfY*) plus 5’ UTR of *cnfY* plus 30 bp of *cnfY* coding region (-82 to +30 bp from ATG of *cnfY*); translational fusion | This study |
| pBO6501 | pFU53; promoter region of YPK_2615 (*cnfY*) plus 5’ UTR of *cnfY* plus 30 bp of *cnfY* coding region (-82 to +30 bp from ATG of *cnfY*), mutant R1 AG32; translational fusion | This study |
| pBO6502 | pFU53; promoter region of YPK_2615 (*cnfY*) plus 5’ UTR of *cnfY* plus 30 bp of *cnfY* coding region (-82 to +30 bp from ATG of *cnfY*), mutant R1+2 ATCAG29-33TCCT; translational fusion | This study |
| pBO6503 | pJNS10-R1; exchange of Gen^R^ cassette in pBO4499 against promoter region of YPK_2615 (*cnfY*) plus 5’ UTR of *cnfY* plus *cnfY* coding region (-82 to +117 bp from *cnfY* ATG), mutant R1 AG32 | This study |
| pBO6504 | pJNS10-R1+2; exchange of Gen^R^ cassette in pBO4499 against promoter region of YPK_2615 (*cnfY*) plus 5’ UTR of *cnfY* plus *cnfY* coding region (-82 to +117 bp from *cnfY* ATG), mutant R1+2 ATCAG29-33TCCT | This study |
| pBO6505 | pBAD2-*gfp*; YPK_2615 (*cnfY*) RNAT plus 30 bp of *cnfY* coding region (-82 to +30 bp from *cnfY* ATG), mutant R1 AG32 33CT | This study |
| pBO6506 | pBAD2-*gfp*; YPK_2615 (*cnfY*) RNAT plus 30 bp of *cnfY* coding region (-82 to +30 bp from *cnfY* ATG), mutant R2 A29Δ | This study |
| pBO6507 | pBAD2-*gfp*; YPK_2615 (*cnfY*) RNAT plus 30 bp of *cnfY* coding region (-82 to +30 bp from *cnfY* ATG), mutant R1+2 ATCAG29-33TCCT | This study |
| pBO6523 | pBAD2-*bgaB*-His; YPK_2615 (*cnfY*) RNAT plus 30 bp of *cnfY* coding region (-82 to +30 bp from *cnfY* ATG), mutant T26C | This study |
| pBO6524 | *Eco*RI(GAATTC) -> *Sac*I(GAGCTC) replacement in pBAD2-*bgaB*-His | This study |
| pBO6527 | pBO6524; 5’-UTR of YPK_2615 (*cnfY*) plus 30 bp of *cnfY* coding region (-266 to +30 bp from *cnfY* ATG) | This study |
| pBO6528 | pBAD2-*bgaB*-His; 5’-UTR of YPK_2615 (*cnfY*) plus 30 bp of *cnfY* coding region (-266 to +30 bp from *cnfY* ATG) | This study |
