# Supplementary material for: An RNA thermometer dictates production of a secreted bacterial toxin
Source: PLoS Pathog. 2020 Jan 17;16(1):e1008184. doi: 10.1371/journal.ppat.1008184 (PMC6992388; doi:10.1371/journal.ppat.1008184)
Supplement: S4 Table — The table includes information about transcriptional regulation of cnfY obtained from previous studies. (DOCX) [file ppat.1008184.s004.docx]

**S4 Table. Transcriptional regulation of *cnfY***

Differential expression in *Y. pseudotuberculosis* strains YPIII (YPK_2615) and IP32953 (YPTB1468; pseudogene). is represented by Log2 fold changes. n.s. = not significant. Data obtained from references [8–11].

| ***Y. pseudotuberculosis* IP32953** | Nuss *et al*., 2017 | *in vivo* vs. 37 °C *in vitro* | | Growth phase | stat | n.s. |
| --- | --- | --- | --- | --- | --- | --- |
|  |  |  |  |  | exp | 1.9 |
|  |  | *in vivo* vs. 25 °C *in vitro* | | Growth phase | stat | n.s. |
|  |  |  |  |  | exp | 2.3 |
| ***Y. pseudotuberculosis* YPIII** | Bücker *et al*., 2014 | 25 °C | Δ*crp* vs. WT | Growth phase | exp | n.s. |
|  |  |  | Δ*csrA* vs. WT | Growth phase | exp | n.s. |
|  |  |  | Δ*rovA* vs. WT | Growth phase | exp | n.s. |
|  | Avican *et al*., 2015 | Aerobic vs anaerobic growth | | Growth phase | stat | 0.8 |
|  |  |  |  |  | exp | 0.9 |
|  |  | Persistent infection vs. early infection | | Growth phase | Not specified | -2.9 |
|  |  | 37 °C *in vitro* vs. 26 °C *in vitro* | | Growth phase | Not specified | 3.0 |
|  | Nuss *et al*., 2015 | Δ*crp* vs. WT | 37 °C | Growth phase | stat | -0.8 |
|  |  |  | 25 °C | Growth phase | stat | 1.5 |
|  |  | 37 °C *in vitro* (Δ *crp*) vs. 25 °C *in vitro* (Δ *crp*) | | Growth phase | stat | n.s |
|  |  | 37 °C *in vitro* vs.  25 °C *in vitro* | | Growth phase | stat | 2.5 |
|  |  |  |  |  | exp | 2.8 |
