# Supplementary figures and images for: An RNA thermometer dictates production of a secreted bacterial toxin
Source: PLoS Pathog. 2020 Jan 17;16(1):e1008184. doi: 10.1371/journal.ppat.1008184 (PMC6992388; doi:10.1371/journal.ppat.1008184)

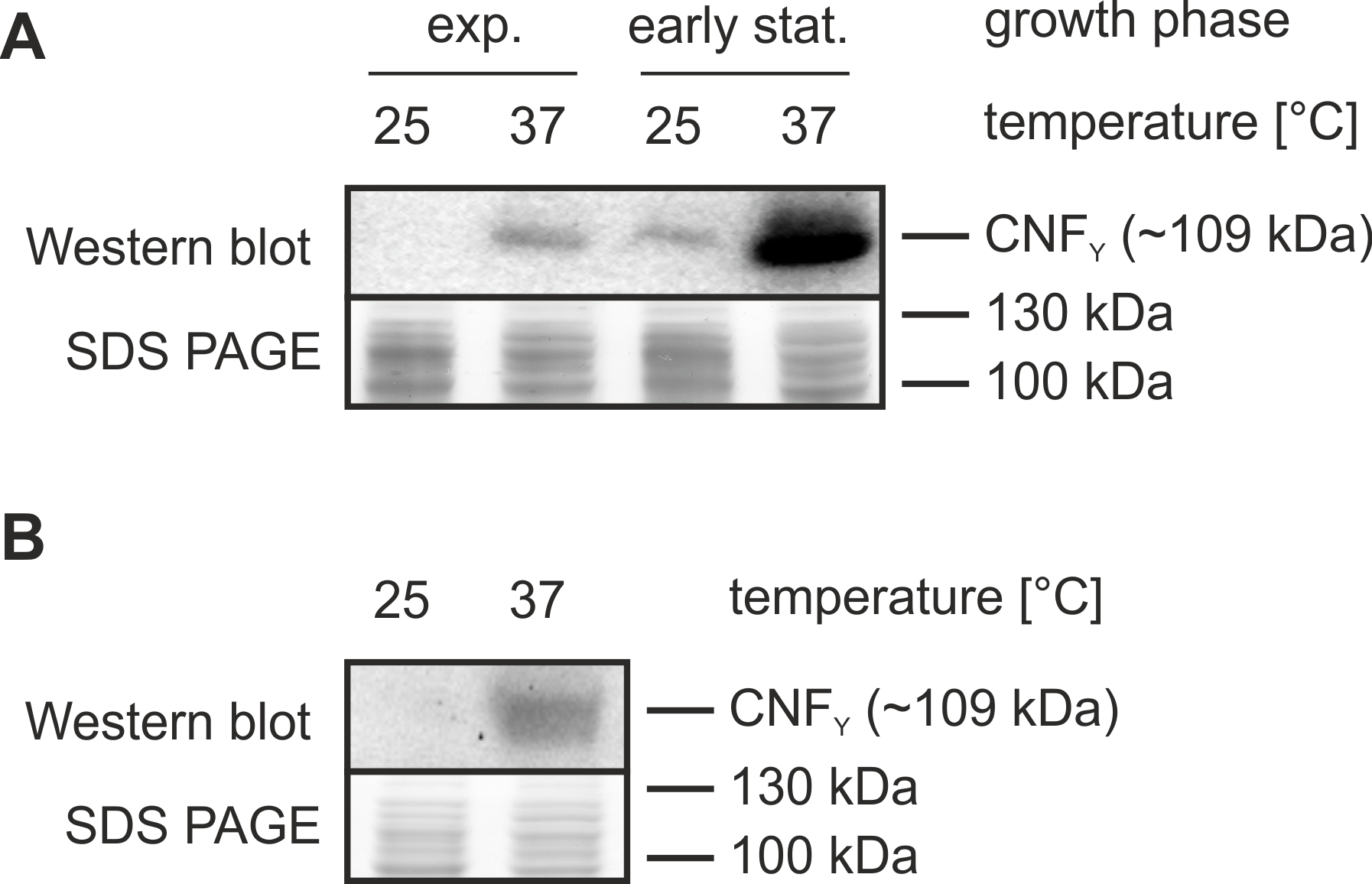

Supplement: S1 Fig — (A) Detection of CNFY protein levels at 25°C and 37°C via Western blot analysis. Y. pseudotuberculosis YPIII cells were grown in LB medium to exponential (OD600 = 0.5) and early stationary growth phase (OD600 = 1.5) and samples were taken for SDS-PAGE and Western blot analysis using a CNFY-specific antibody. (B) Representative, original Western blot for CNFY quantification at 25°C and 37°C used for data analysis displayed in Fig 1A. Samples were taken from a Y. pseudotuberculosis YPIII culture grown in LB medium to exponential growth phase (OD600 = 0.5). (TIF) [file ppat.1008184.s005.tif]

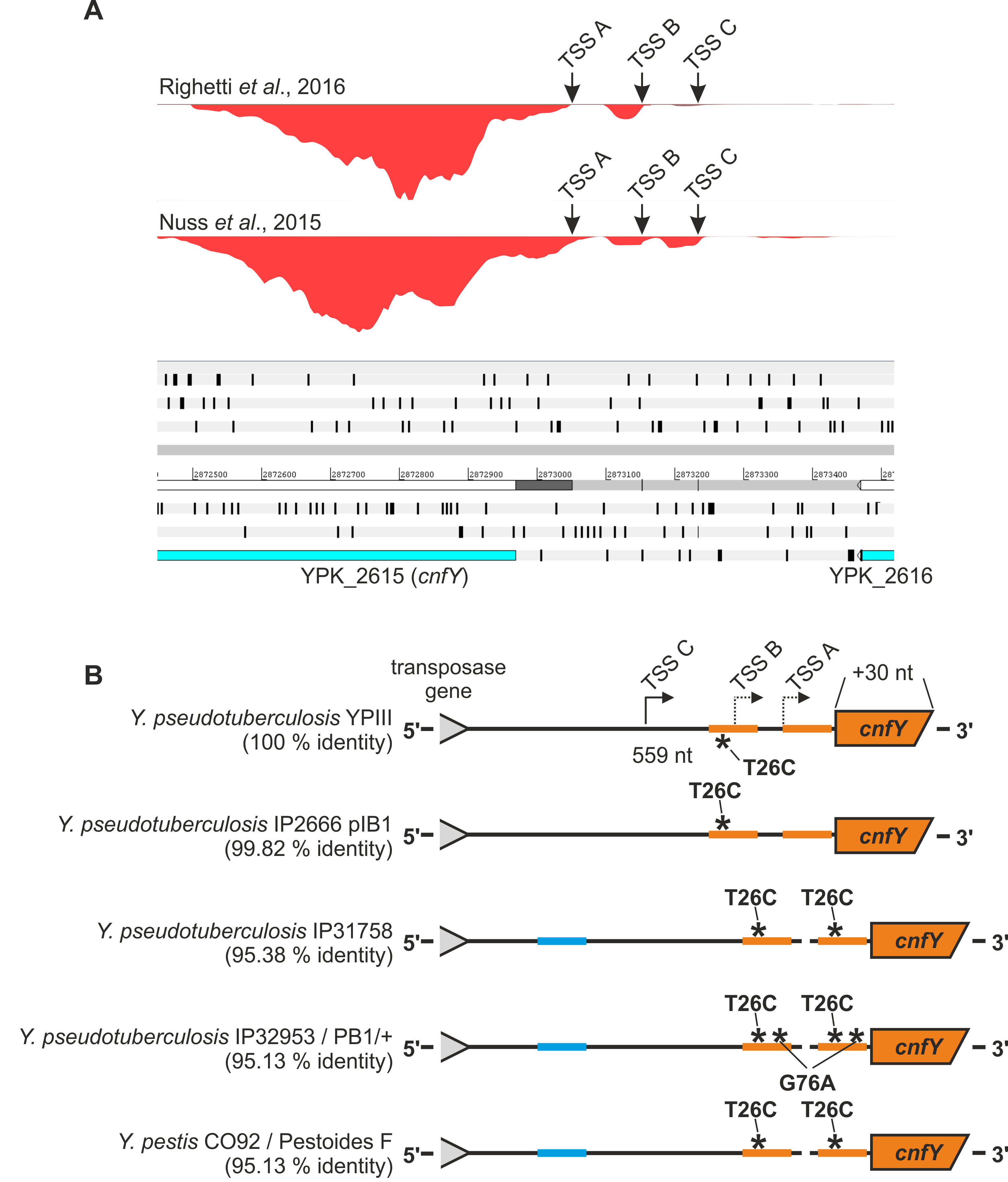

Supplement: S2 Fig — (A) Representation of RNA-seq (37°C) based cDNA reads according to Righetti et al., 2016 [6] and Nuss et al., 2015 [10] visualized via the Artemis genome browser [12]. Different potential TSSs (for details see Fig 1A) are indicated by arrows. (B) Schematic representation of the cnfY upstream region (559 nt) including 30 nt of the cnfY coding region and 29 nt of the upstream transposase gene found in Y. pseudotuberculosis strains YPIII (GenBank accession: CP009792), IP2666 pIB1 (CP032566), IP31758 (CP000720), IP32953 (CP009712), and PB1/+ (CP009780) or in Y. pestis strains CO92 (CP009973) and Pestoides F (CP00668). The cnfY RNAT sequence and its upstream duplication are marked in orange. Sequence insertions are marked in blue, whereas asterisks mark nucleotide exchanges within the RNAT sequences (relative to Y. pseudotuberculosis YPIII). Broken lines indicate sequence deletions relative to the Y. pseudotuberculosis YPIII genome. Overall sequence identities (relative to Y. pseudotuberculosis YPIII) are displayed under each species name. (TIF) [file ppat.1008184.s006.tif]

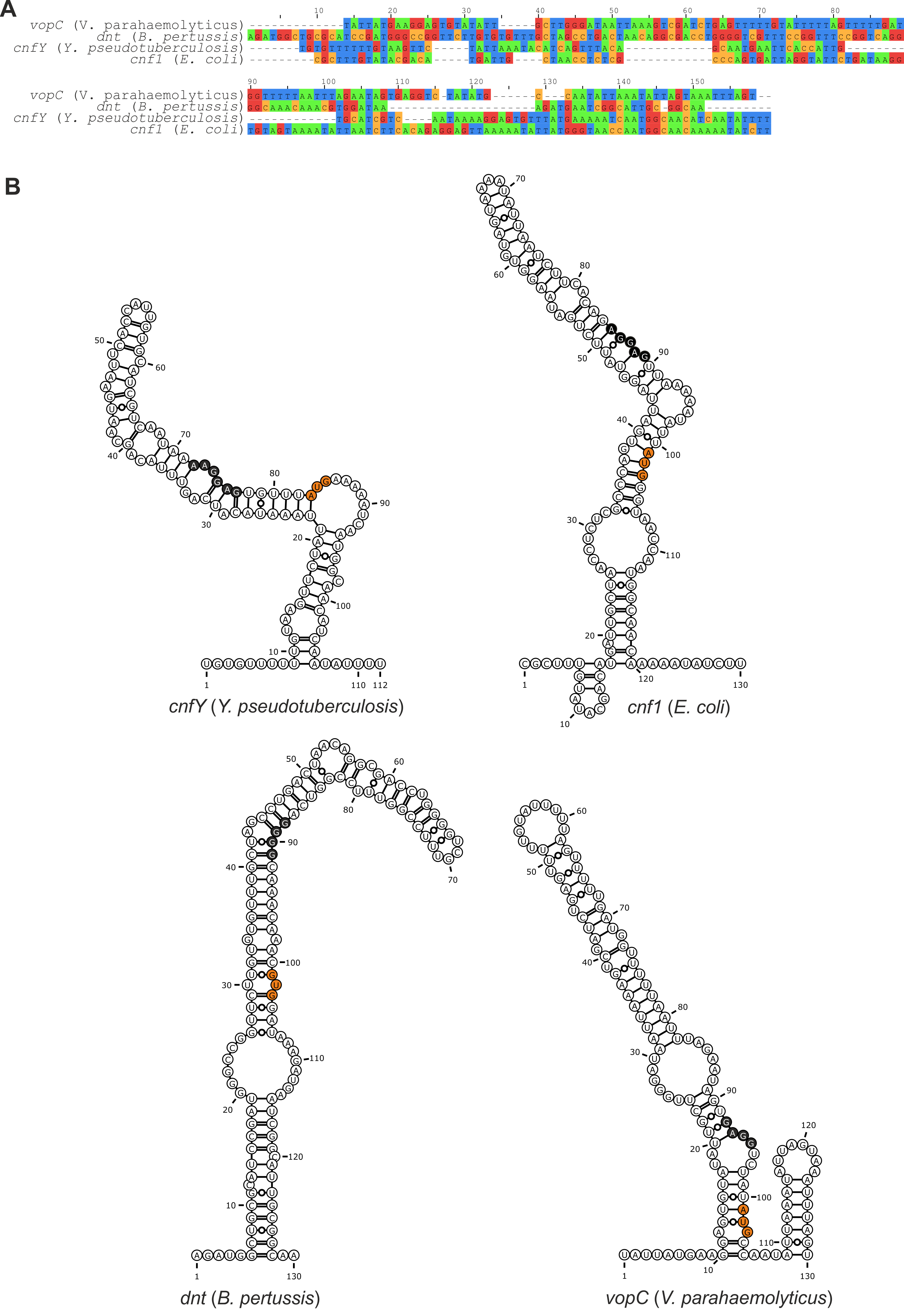

Supplement: S3 Fig — (A) Multiple alignment of sequences located upstream of genes coding for CNFs or related toxins. Displayed is the alignment of sequences upstream of cnfY from Y. pseudotuberculosis YPIII, cnf1 from E. coli O18:K1:H7 UTI89, dnt from Bordetella pertussis J262, and vopC from Vibrio parahaemolyticus BB22OP. The multiple sequence alignment was calculated with Clustal Omega (https://www.ebi.ac.uk/Tools/msa/clustalo/) and visualized via jalview application [13]. (B) Secondary structures of the cnfY RNAT (-82 nt; [6]) from Y. pseudotuberculosis YPIII (ΔG° = -13.19; [ΔG°] = kcal*mol-1) and the upstream regions (-100 nt and +30 nt from AUG) of cnf1 from E. coli O18:K1:H7 UTI89 (ΔG° = -28.20), dnt from B. pertussis J262 (ΔG° = -61.93) and vopC from V. parahaemolyticus BB22OP (ΔG° = -23.05) are displayed. Structure of the cnfY RNAT originates from [6]. The remaining structures were predicted via RNAfold [14] with temperature set to 25°C. The proposed SD sequences and AUG start codon are depicted in black and orange, respectively. (TIF) [file ppat.1008184.s007.tif]
