# Supplementary material for: An RNA thermometer dictates production of a secreted bacterial toxin
Source: PLoS Pathog. 2020 Jan 17;16(1):e1008184. doi: 10.1371/journal.ppat.1008184 (PMC6992388; doi:10.1371/journal.ppat.1008184)
Supplement: S1 References — (DOCX) [file ppat.1008184.s008.docx]

# S1 References. References for Supporting Information

1. Bölin I, Norlander L, Wolf-Watz H. Temperature-inducible outer membrane protein of *Yersinia pseudotuberculosis* and *Yersinia enterocolitica* is associated with the virulence plasmid. Infect Immun. 1982; 37(2):506–12.PMID: 6749681

2. Schweer J, Kulkarni D, Kochut A, Pezoldt J, Pisano F, Pils MC, Genth H, Huehn J, Dersch P. The Cytotoxic necrotizing factor of *Yersinia pseudotuberculosis* (CNF_Y_) enhances inflammation and Yop delivery during infection by activation of Rho GTPases. PLoS Pathog. 2013; 9(11):e1003746. doi: 10.1371/journal.ppat.1003746 PMID: 24244167

3. Hanahan D. Studies on transformation of *Escherichia coli* with plasmids. J Mol Biol. 1983; 166(4):557–80. doi: 10.1016/S0022-2836(83)80284-8 PMID: 6345791

4. Yanisch-Perron C, Vieira J, Messing J. Improved Ml3 phage cloning vectors and host strains: nucleotide sequences of the M13mp18 and pUC19 vectors. Gene. 1985; 33(1985):103–19. doi: 10.1016/0378-1119(85)90120-9 PMID: 2985470

5. Roßmanith J, Narberhaus F. Exploring the modular nature of riboswitches and RNA thermometers. Nucleic Acids Res. 2016; 44(11):5410–23. doi: 10.1093/nar/gkw232 PMID: 27060146

6. Righetti F, Nuss AM, Twittenhoff C, Beele S, Urban K, Will S, Bernhart SH, Stadler PF, Dersch P, Narberhaus F. Temperature-responsive *in vitro* RNA structurome of *Yersinia pseudotuberculosis*. Proc Natl Acad Sci USA. 2016; 113(26):7237–42. doi: 10.1073/pnas.1523004113 PMID: 27298343

7. Uliczka F, Pisano F, Kochut A, Opitz W, Herbst K, Stolz T, Dersch P. Monitoring of gene expression in bacteria during infections using an adaptable set of bioluminescent, fluorescent and colorigenic fusion vectors. PLoS One. 2011; 6(6):e20425. doi: 10.1371/journal.pone.0020425 PMID: 21673990

8. Avican K, Fahlgren A, Huss M, Heroven AK, Beckstette M, Dersch P, Fällman M. Reprogramming of *Yersinia* from virulent to persistent mode revealed by complex *in vivo* RNA-seq analysis. PLoS Pathog. 2015; 11(1):1–28. doi: 10.1371/journal.ppat.1004600 PMID: 25590628

9. Nuss AM, Beckstette M, Pimenova M, Schmühl C, Opitz W, Pisano F, Heroven AK, Dersch P. Tissue dual RNA-seq allows fast discovery of infection-specific functions and riboregulators shaping host–pathogen transcriptomes. Proc Natl Acad Sci USA. 2017; 114(5):E791–800. doi: 10.1073/pnas.1613405114 PMID: 28096329

10. Nuss AM, Heroven AK, Waldmann B, Reinkensmeier J, Jarek M, Beckstette M, Dersch P. Transcriptomic profiling of *Yersinia pseudotuberculosis* reveals reprogramming of the Crp regulon by temperature and uncovers Crp as a master regulator of small RNAs. PLoS Genet. 2015; 11(3):e1005087. doi: 10.1371/journal.pgen.1005087 PMID: 25816203

11. Bücker R, Heroven AK, Becker J, Dersch P, Wittmann C. The pyruvate-tricarboxylic acid cycle node: a focal point of virulence control in the enteric pathogen *Yersinia pseudotuberculosis*. J Biol Chem. 2014; 289(43):30114–32. doi: 10.1074/jbc.m114.581348 PMID: 25164818

12. Carver T, Harris SR, Berriman M, Parkhill J, McQuillan JA. Artemis: An integrated platform for visualization and analysis of high-throughput sequence-based experimental data. Bioinformatics. 2012; 28(4):464–9. doi: 10.1093/bioinformatics/btr703 PMID: 22199388

13. Clamp M, Cuff J, Searle SM, Barton GJ. The Jalview Java alignment editor. Bioinformatics. 2004; 20(3):426–7. doi: 10.1093/bioinformatics/btg430 PMID: 14960472

14. Lorenz R, Bernhart SH, zu Siederdissen C, Tafer H, Flamm C, Stadler PF, Hofacker IL. ViennaRNA Package 2.0. Algorithms Mol Biol. 2011; 6(1):26. doi: 10.1186/1748-7188-6-26 PMID: 22115189
